# Mitochondrial Sensitivity to Submaximal [ADP] Following Bed Rest: A Novel Two‐Phase Approach Associated With Fibre Types

**DOI:** 10.1002/jcsm.13775

**Published:** 2025-04-25

**Authors:** Lucrezia Zuccarelli, Maria De Martino, Antonio Filippi, Alice E. Knapton, Benjamin D. Thackray, Giovanni Baldassarre, Boštjan Šimunič, Rado Pišot, Giuseppe Sirago, Elena Monti, Marco Narici, Miriam Isola, Andrew J. Murray, Giovanna Lippe, Bruno Grassi

**Affiliations:** ^1^ Department of Medicine University of Udine Udine Italy; ^2^ Department of Physiology, Neuroscience and Development University of Cambridge Cambridge UK; ^3^ Institute of Kinesiology Research Science and Research Centre Koper Slovenia; ^4^ Department of Biomedical Sciences University of Padova Padova Italy; ^5^ Institute of Sport Sciences and Department of Biomedical Sciences University of Lausanne Lausanne Switzerland

**Keywords:** ADP, bed rest, mitochondrial sensitivity, myosin heavy chains, skeletal muscle mitochondria

## Abstract

**Background:**

We recently demonstrated that following a 10‐day exposure to inactivity/simulated microgravity impairments of oxidative metabolism were located ‘upstream’ of mitochondrial function, as evaluated by maximal ADP‐stimulated mitochondrial respiration (JO_2max_) determined ex vivo. The aim of this study was to evaluate mitochondrial sensitivity to submaximal [ADP] by an alternative approach aimed at identifying responses associated with fibre type composition.

**Methods:**

Isolated permeabilized *vastus lateralis* fibres were analysed by high‐resolution respirometry in 9 young males before and after a 10‐day horizontal bed rest. Eleven submaximal titrations of ADP (from 12.5 to 10 000 μM) were utilized to assess complex I + II‐linked ADP sensitivity. We applied to JO_2_ versus [ADP] data a traditional Michaelis–Menten kinetics equation, with the calculation of the apparent K_m_ and maximal respiration (V_max_), and two ‘sequential’ hyperbolic equations, yielding two K_m_ and V_max_ values. The two‐hyperbolic equations were solved and the [ADP] value corresponding to 50% of JO_2max_ was calculated. Isoform expression of myosin heavy chains (MyHC) 1, 2A and 2X was also determined. Control experiments were also carried out on rat skeletal muscle samples with different percentages of MyHC isoforms.

**Results:**

The two hyperbolic equations provided an alternative fitting of data and identified two distinct phases of the JO_2_ versus [ADP] response: a first phase characterized by low V_max_ (V_max1_, 28 ± 10 pmol s^−1^ mg^−1^) and apparent K_m_ (K_m1_, 62 ± 54 μM) and a second phase characterized by higher V_max_ (V_max2_, 61 ± 16 pmol s^−1^ mg^−1^) and K_m_ (K_m2_, 1784 ± 833 μM). Data were confirmed in control experiments carried out in rat muscle samples with different percentages of MyHC isoforms. Correlation and receiver operating characteristics analyses suggest that the two phases of the response were related to the % of MyHC isoforms.

**Conclusions:**

A novel mathematical approach (two sequential hyperbolic functions) for the fitting of JO_2_ versus [ADP] data obtained by high‐resolution respirometry on permeabilized skeletal muscle fibres, obtained in humans and rats, provided an alternative fitting of the experimental data compared to the traditional Michaelis–Menten kinetics equation. This alternative model allowed the identification of two distinct phases in the responses, which were related to fibre type composition. A first phase, characterized by low apparent K_m_ and V_max_ values, was correlated with the percentage of less oxidative (Type 2A + 2X) MyHC isoforms. A second phase, characterized by high apparent K_m_ and V_max_, was related to more oxidative (Type 1) MyHC isoforms.

## 1 Introduction

The main sites of impairment of oxidative metabolism during exercise following a 10‐day exposure to inactivity/simulated microgravity (horizontal bed rest) were recently demonstrated to be upstream of mitochondria [1, 2]. Impairments of peak oxygen uptake (V̇O_2_peak), peak cardiac output [2] and microvascular/endothelial functions [1] were indeed observed following a 10‐day bed rest. In contrast, indices of mitochondrial function, such as maximal ADP‐stimulated mitochondrial respiration ex vivo in isolated permeabilized fibres, and muscle V̇O_2_ off‐kinetics evaluated in vivo by near‐infrared spectroscopy, were unaffected [1]. Citrate synthase (CS) activity, taken as an index of mitochondrial mass, was also unaffected [1].

Besides being the primary energy producers of the cells via oxidative phosphorylation, mitochondria support many important cellular functions including metabolic homeostasis, apoptosis and redox balance [3, 4]. The biological importance of mitochondrial function (or dysfunction/shift in functional demand) is indicated by the links with exercise performance, diseases and mortality [5, 6, 7, 8, 9, 10]. Given the variability in defining ‘mitochondrial function’ and ‘dysfunction’ across studies, we emphasize that in this study, ‘function’ simply refers to mitochondrial respiration. Interestingly, however, in some studies performed on patients with Type 2 diabetes [11, 12], in elderly subjects [13, 14] or in subjects exposed to short‐term simulated microgravity [1, 15, 16], conditions in which oxidative metabolism is known to be impaired, maximal ADP‐stimulated mitochondrial respiration was not altered. This variable, although representing an index of maximal mitochondrial respiratory capacity, involves the utilization of [ADP] (squared brackets denote concentrations), which are orders of magnitude greater than ‘physiological’ free [ADP] (25–250 μM [17, 18]). According to some authors [4], analysis of the sensitivity of mitochondrial respiration to submaximal [ADP] would represent another valuable tool for the functional evaluation of mitochondrial respiration.

This sensitivity is commonly evaluated ex vivo (e.g. in permeabilized skeletal muscle fibres [19]) by high‐resolution respirometry and by performing several submaximal [ADP] titrations, some of which correspond to the physiological range. The Michalis–Menten (MM) kinetics curve is traditionally utilized as the model to fit mitochondrial oxygen flux (JO_2_) versus [ADP] [20]. The variables evaluated by the MM kinetics are V_max_, which represents the maximal respiration rate, and the apparent K_m_, which evaluates the sensitivity of mitochondrial respiration to [ADP], as the [ADP] needed to support 50% of V_max_.

Fitting by the MM kinetics curve, however, is at times not optimal, with overestimation of experimental data by the fitting function at low [ADP] and underestimation at higher [ADP] [14, 21]. Nonetheless, the vast majority of previous studies carried out on this topic did not explore alternative models to fit JO_2_ versus [ADP] data. Some early studies, mainly conducted on animal models, reported a deviation from the MM kinetics model [20, 22, 23, 24, 25], with two distinct phases of the JO_2_ versus [ADP] kinetics [20, 23]. Each phase being characterized by distinct V_max_ and K_m_ values. Studies of rodent models [23] reported tissue specificity of mitochondrial affinity for ADP. Highly oxidative muscles, such as slow‐twitch soleus skeletal muscle and heart, displayed a very low affinity for ADP (very high K_m_ values) and high mitochondrial respiration rates, whereas in fast‐twitch muscles, such as gastrocnemius and tibialis anterior, the affinity to ADP was very high (very low K_m_) [23].

The present study aimed to re‐examine, in skeletal muscle biopsies obtained from the participants of a 10‐day bed rest [1], data on the mitochondrial sensitivity to submaximal [ADP] by applying a novel mathematical model characterized by two phases and to compare the results with the widely utilized MM kinetics equation. We further sought evidence supporting the concept that the two‐phase model correlates with fibre type composition by looking at correlations with the myosin heavy chain (MyHC) composition of the fibres. Confirmatory experiments were carried out on rat skeletal muscle samples with different percentages of MyHC isoforms.

## 2 Methods

### 2.1 Ethical Approval

This study was part of the Italian Space Agency (ASI) project ‘MARS‐PRE Bed Rest SBI 2019’. It was approved by the National Ethical Committee of the Slovenian Ministry of Health (ref. number: 0120‐304/2019/9) and was performed in accordance with the standard set by the *Declaration of Helsinki*. All participants were informed about the aims, procedures and potential risks of the investigations before written consent was obtained. All procedures involving live animals were carried out by a licence holder in accordance with UK Home Office regulations and underwent review by the University of Cambridge Animal Welfare and Ethical Review Committee.

### 2.2 Subjects

The experimental set‐up has been previously described in detail [1]. The studies examined nine healthy recreationally active men. The main physical characteristics at baseline were age 23 ± 5 years; height 1.81 ± 0.04 m; body mass 78 ± 10.5 kg; body mass index 23.7 ± 2.5 kg m^−2^. Each subject was evaluated before and after 10 days of strict horizontal bed rest without countermeasures. The experiments were carried out at the Izola General Hospital, Slovenia. The protocol included 3 days of familiarization with the study environment, pre–bed rest data collection (PRE), 10 days of bed rest and post–bed rest (POST) data collection. During bed rest, no deviations from the lying position, muscle stretching or static contraction were allowed. The dietary energy requirement was designed for each subject by multiplying resting energy expenditure by factors 1.2 and 1.4 during the bed rest and ambulatory periods, respectively [26]. The macronutrient food content was set at 60% carbohydrate, 25% fat and 15% protein. Subjects were allowed to drink water *ad libitum*.

### 2.3 Animal Care

Male Wistar rats (Charles River Laboratories) 270–300 g on arrival (*n* = 4) were pair‐housed in conventional cages in a temperature (23°C) and humidity‐controlled environment with a 12‐h/12‐h light/dark cycle. Rats were fed a standard diet (RM1(P), Special Diet Services, UK) and had access to food and water *ad libitum*. Body mass, food and water intake were measured daily. After 26–27 days, anaesthesia was induced in rats by inhalation of isofluorane (5% in 100% O_2_). Following cessation of peripheral signs, rats received an i.p. injection of pentobarbital (Euthatal, Merial) at a dose of 500 mg kg^−1^ body mass. After confirmation of death, skeletal muscles (soleus and tibialis anterior) were rapidly excised and placed on ice‐cold biopsy preservation medium for the analysis of mitochondrial respiratory function, as described for human skeletal muscle biopsies [1].

### 2.4 Mitochondrial Respiration

Skeletal muscle biopsies were obtained from *vastus lateralis* muscle under local anaesthesia (2% lidocaine, 2 mL) for the evaluation of mitochondrial respiration by high‐resolution respirometry and fibre type composition, during Day 1 and Day 10 of the bed rest. The biopsies were obtained from participants in the early morning, shortly after breakfast.

All analysis methods are described in detail in a previous work [1]. Briefly, following the application of the anaesthetic, a 1.0–1.5 cm incision was made to the skin, subcutaneous tissue and muscle fascia, and the tissue sample was harvested with a Rongeur‐Conchotome (GmbH & Co., Zepf Instruments, Dürbheim, Germany). Muscle tissue samples were dissected to remove fat and connective tissue and quickly divided into several portions. A small portion (2.0–6.5 mg wet weight) was immediately used to evaluate mitochondrial respiration ex vivo, with measurements performed in duplicate. The tissue was placed in an ice‐cold preservation solution (BIOPS) containing various buffers and compounds [27] at 4°C. Fibre bundles were trimmed and separated under magnification and incubated in BIOPS for 10 min and then in a BIOPS solution containing 50 μg mL^−1^ saponin for 30 min at 4°C to ensure permeabilization. The samples were washed once for 10 min with a respiration medium (MIR05), weighed and transferred to a respirometer (Oxygraph‐2k) chamber for high‐resolution respirometry to measure O₂ consumption. The experiments were conducted at 37°C in an air‐saturated respiration medium (MIR06) to avoid oxygen limitation with the myosin II‐ATPase inhibitor Blebbistatin (25 μM, dissolved in DMSO 5 mM stock) included to prevent spontaneous contraction [28]. The O_2_ concentration in the chamber was maintained between 280 and 400 μM (average O_2_ partial pressure 250 mmHg) to avoid O_2_ limitation of respiration. The chamber lights were kept on during the experiments.

In order to evaluate the sensitivity of JO_2_ to submaximal [ADP] [14], JO_2_ was determined, in the presence of glutamate (10 mM), malate (4 mM) and succinate (4 mM), following the administration of 11 increasing [ADP] (i.e. 12.5, 25, 175, 250, 500, 1000, 2000, 4000, 6000, 8000 and 10 000 μM) (Figure S1). JO_2_ values (expressed as a percentage of JO_2max_) were fitted by a *traditional MM kinetics equation*:

(1) $$y=\frac{V_{\max}x}{\;K_{m}+x}$$

where *x* is [ADP] (μM), *y* indicates the JO_2_ at the given [ADP], V_max_ is the maximal JO_2_ and the apparent *K*_m_ is the [ADP] providing 50% of V_max_.

Data were also fitted by two ‘superimposed’ or ‘sequential’ hyperbolic equations, each characterized by different apparent K_m_ and V_max_ values. *Superimposed hyperbolic equations* were originally proposed by Saks et al. [20]:

(2) $$y=\frac{\;{V_{\max}}_{1}x}{K_{m1}+x}+\frac{{V_{\max}}_{2}x}{K_{m2}+x}$$

in which *x* is [ADP] (μM), *y* indicates JO_2_ at the given [ADP], V_max1_ and V_max2_ are the JO_2max_ of the first and the second superimposed hyperbolic functions, respectively, and K_m1_ and K_m2_ are the [ADP] needed to stimulate 50% of V_max1_ and V_max2_, respectively.

Data were also fitted by maximizing two *sequential hyperbolic equations*:

(3) $$y=\max\left\{\frac{\;{V_{\max}}_{1}x}{K_{m1}+x},\frac{{V_{\max}}_{2}x}{K_{m2}+x}\right\}$$

in which *x* is [ADP] (μM); *y* indicates JO_2_ at the given [ADP], V_max1_ and V_max2_ are the JO_2max_ of the first and the second sequential hyperbolic functions, respectively; and K_m1_ and K_m2_ are the [ADP] needed to stimulate 50% of V_max1_ and V_max2_, respectively.

A variable equivalent to the apparent K_m_ (see above) of the traditional MM kinetics equation was then calculated by solving equation 2 and 3 in order to determine the [ADP] corresponding to 50% of the overall JO_2_ response ([ADP] at 50% of JO_2max_).

### 2.5 Analysis of MyHC Isoforms by SDS‐PAGE

Relative content of MyHC isoforms in skeletal muscle samples from rats was evaluated as [29], but the data for humans were directly obtained from the reference [29]. Briefly, 5–10 mg of biopsy was solubilized in Laemmli solution (62.5 mM Tris–HCl pH 6.8, 2.3% SDS, 10% glycerol) [30], treated with one cycle of thermal shock at 65°C for 3 min and then stored at −20°C to allow the complete resuspension of proteins and breaking of membranes. Protein concentration was determined by the Folin‐Lowry method, using BSA as standard [31]. Proteins from each sample (∼8 μg) were separated on 8% SDS‐PAGE with custom‐made mini‐gels (Mini‐PROTEAN Tetra Handcast System, Bio‐Rad) and electrophoresis was run in a cold room for 1 h at 50 V constant and then for ∼40 h at 60 V constant. Afterwards, the gel was stained with the Colloidal Coomassie Blue Staining method, modified from previous protocols [32]. Protein bands (MyHC‐1, MyHC‐2A and MyHC‐2X) were quantified by densitometric analysis (Li‐Cor, Odyssey CLx, Image Studio Ver. 5.2) to assess the relative proportion of each myosin isoform within each subject. The same method was used to determine the MyHC isoform distributions in rats. In the gels, three bands were detectable in the region between 200 and 250 kDa corresponding (from the fastest to the slowest migrating band) to MyHC‐1, MyHC‐2B and MyHC‐2X/2A.

### 2.6 Statistical Analysis

Results are expressed as mean ± SD values. Continuous variables were compared using a Student *t*‐test or Mann–Whitney *U* test for two groups, or by one‐way ANOVA or the Kruskal–Wallis test for more than two groups, according to the Shapiro–Wilk test. Data fitting with the different models was performed by the least‐squared residuals method. Comparisons between fitting with different models were carried out by considering the residual sum of squares (RSS) and *r* ^2^. De Long's non‐parametric receiver operating characteristic (ROC) analysis was conducted to investigate the capacity, by the percentage of Type 2 (2A and 2X) muscle fibre myosin heavy chain isoforms (MyHC 2A + 2X), to discriminate if the value corresponding to the apparent K_m_ value ([ADP] at 50% of JO_2max_) occurred in the [ADP] domain corresponding to the first hyperbolic function. Hence, ROC analysis was utilized to identify a cut‐off probability for MyHC 2A + 2X fibres to discriminate when the value of [ADP] at 50% of JO_2max_ occurred in the domain of the first sequential hyperbolic function. The level of significance was set at *p* < 0.05. Statistical analyses were carried out by commercially available software packages (Prism 9, GraphPad and STATA 17).

## 3 Results

### 3.1 Data Obtained on Humans Before and After Bed Rest

Data related to anthropometric characteristics, systemic and peripheral variables evaluating oxidative metabolism and maximal mitochondrial respiration rates are presented in two previous papers by our group [1, 2]. Maximal O_2_ uptake values were 45.4 ± 7.0 mL kg^−1^ min^−1^ in PRE and 40.9 ± 6.1 in POST (*p* = 0.001) [2].

**FIGURE 1:**
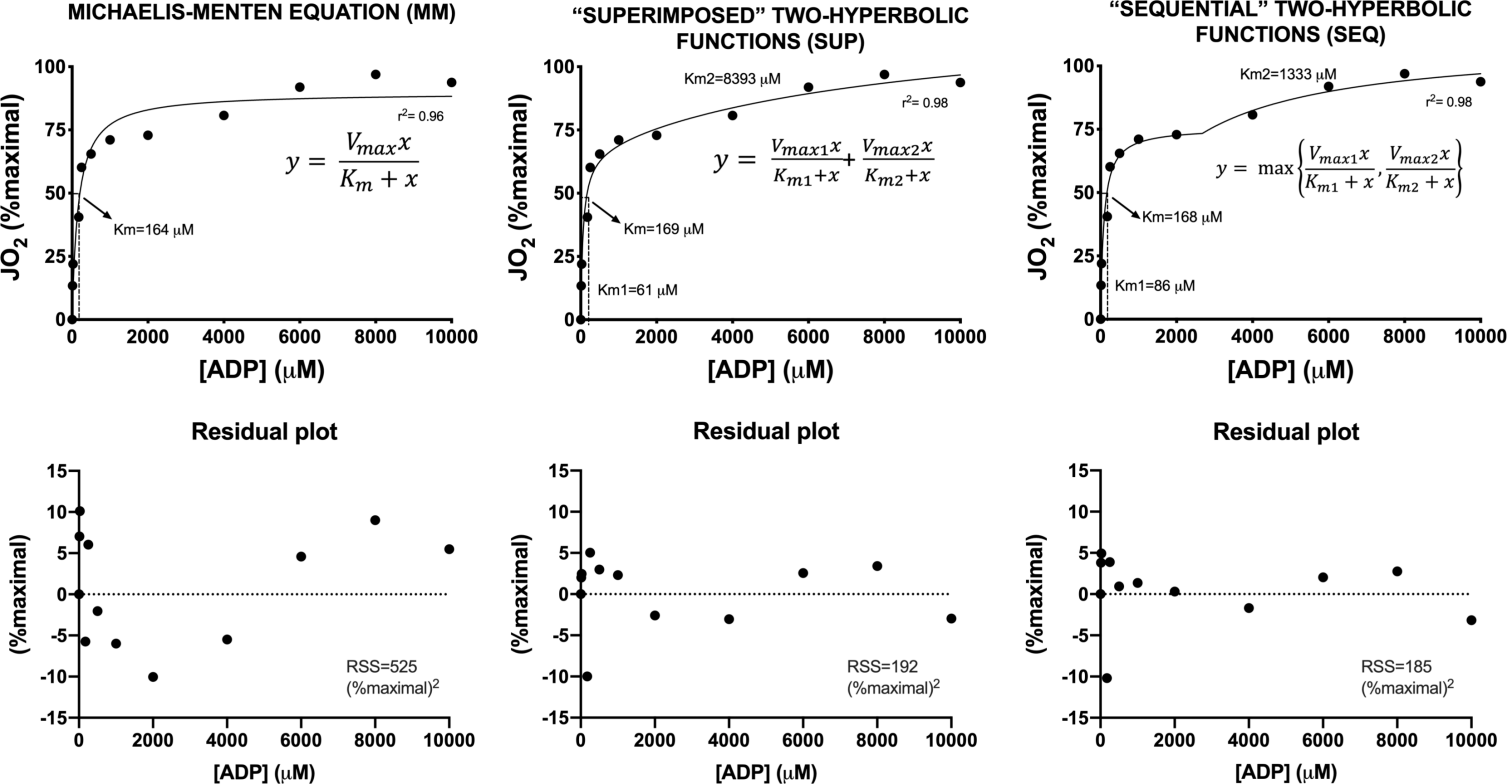
ADP‐stimulated mitochondrial respiration. In the upper panels, respiration rates (JO_2_, expressed as a percentage of maximal values) as a function of [ADP] in a typical subject PRE bed rest are shown. Data were fitted using three different mathematical models (see Section 2 and equations in the graphs). K_m_ indicates the [ADP] at 50% of JO_2max_; K_m1_ and K_m2_ are the [ADP] values needed to stimulate the 50% of V_max1_ and V_max2_, respectively. In the lower panels, analysis of residuals showed an increased quality of the fitting for the superimposed and for the sequential two‐hyperbolic functions compared to the traditional MM kinetics equation. Goodness of fittings was evaluated by the *r* ^2^ and the residual sum of squares (RSS) values. See text for further details.

**FIGURE 2:**
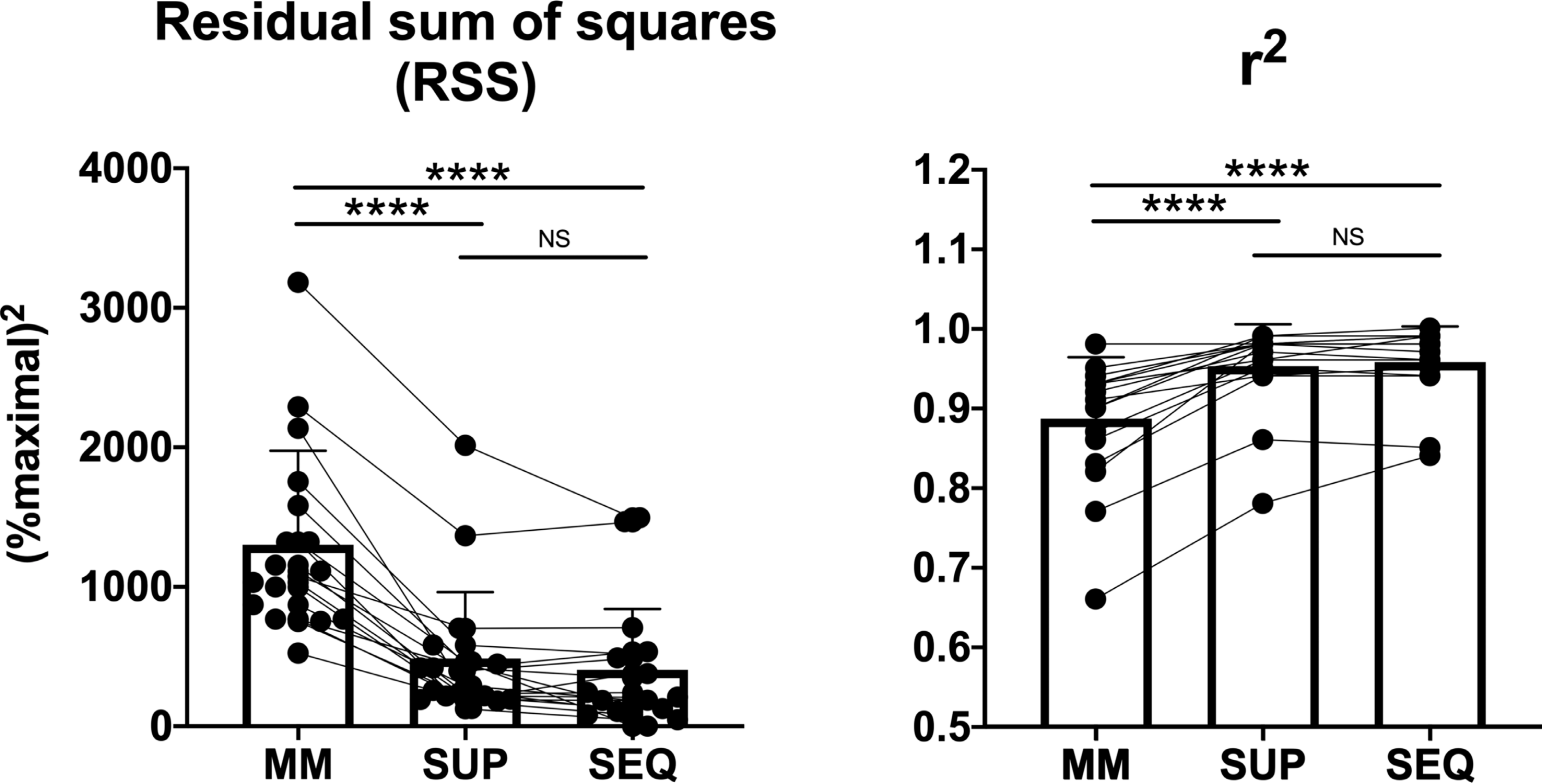
Individual and mean (±SD) values of the sum of squared residuals (*r* ^2^) are shown in the graphs for the three different fitting approaches (Michaelis–Menten equation [MM], superimposed [SUP] and sequential [SEQ] two‐hyperbolic functions). See text for further details. NS, not significant; ****p < 0.0001.

Individual oxygraph traces (*n* = 52) of ADP‐stimulated mitochondrial respiration in permeabilized skeletal muscle fibres are given in Figure S2. JO_2_ (expressed as a percentage of JO_2max_) versus [ADP] data were analysed according to three different equations (see Section 2). Figure 1 (upper panels) shows typical individual examples of the fittings obtained on one subject before bed rest: the traditional MM kinetics equation (left panel), the superimposed (middle panel) and the sequential (right panel) two‐hyperbolic equations. Absolute mitochondrial oxygen consumption versus [ADP] data are shown in Figure S3. The quality of the fitting increased with the two‐hyperbolic approaches compared with MM kinetics. More specifically, the MM kinetics equation markedly overestimated JO_2_ from about 1000 to about 4000 μM and underestimated JO_2_ from about 6000 to about 10 000 μM. This was confirmed by the analysis of residuals (lower panels of Figure 1). The quality of fitting and residual plots were not different between PRE and POST. The higher quality of the fitting by the two‐hyperbolic approaches was also confirmed by analysing the sum of squared residuals and the *r* ^2^ values, as shown in Figure 2.

The two sequential hyperbolic fittings identified two distinct JO_2_ versus [ADP] phases. A first phase, characterized by low K_m1_ and V_max1_ values, was indeed followed, for [ADP] values higher than about 3000 μM, by a second phase characterized by high K_m2_ and V_max2_ values.

**FIGURE 3:**
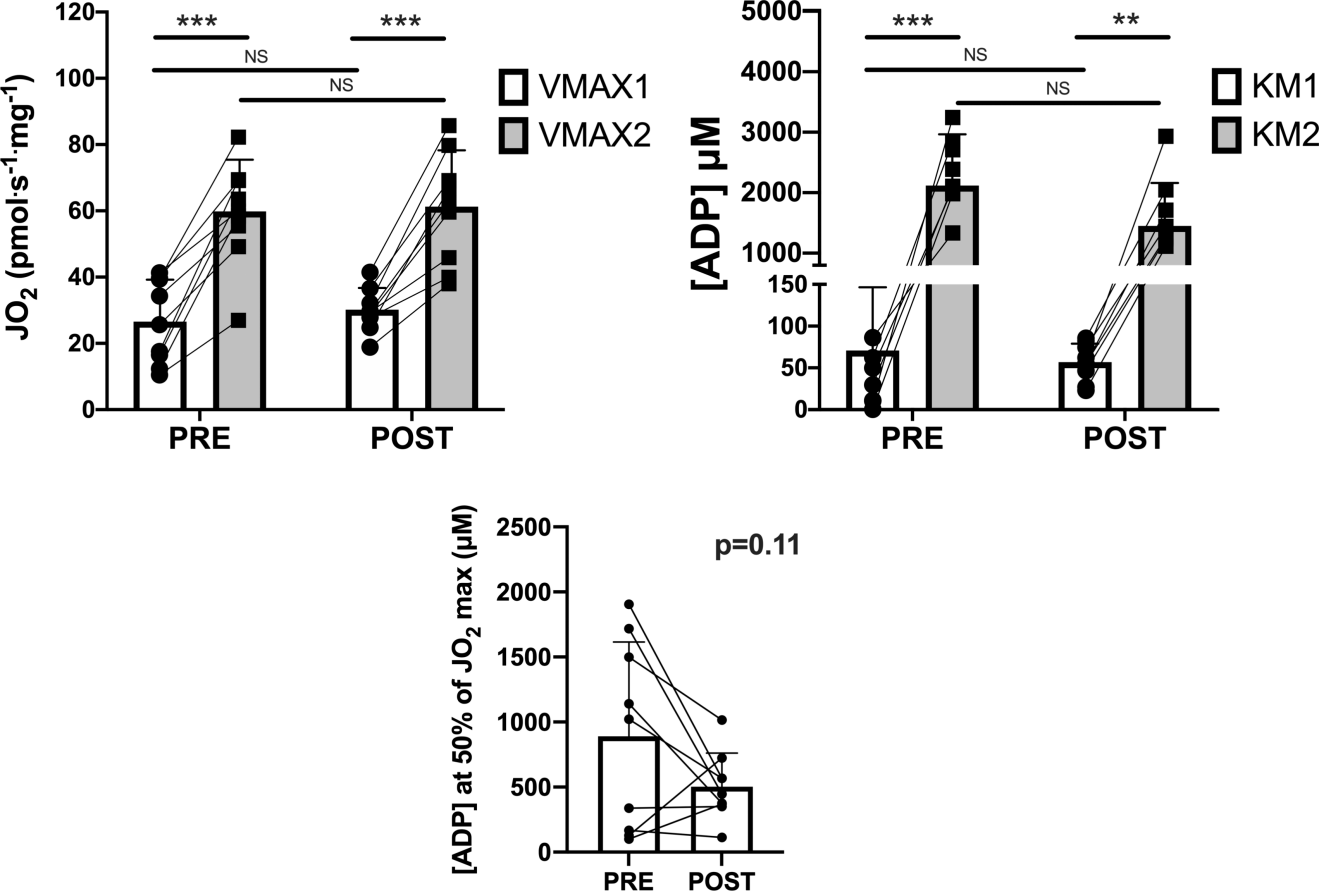
Individual and mean (± SD) values of V_max1_, V_max2_, K_m1_ and K_m2_, obtained before (PRE) and after (POST) 10‐day horizontal bed rest by the sequential two‐hyperbolic function fitting approach are shown. Individual and mean (± SD) [ADP] values corresponding to the apparent K_m_ for a MM kinetics ([ADP] at 50% of JO_2max_) are also shown. See text for further details. NS, not significant; ***p < 0.001; **p < 0.01.

V_max1_ and V_max2_ values obtained by the sequential two‐hyperbolic approach are shown in Figure 3. V_max1_ was significantly lower than V_max2_ both in PRE and POST (*p* < 0.001). Both V_max1_ and V_max2_ were not significantly different in POST versus PRE (*p* = 0.95 and *p* = 0.99, respectively) (see Figure 3, left upper panel). K_m1_ values were significantly (about 30 times) lower than K_m2_ values, both in PRE and POST. K_m1_ was not significantly different in POST versus PRE (*p* = 0.60), whereas a trend for K_m2_ to decrease was observed in POST versus PRE (*p* = 0.16) (see Figure 3, right upper panel).

The [ADP] value corresponding, by solving the sequential two‐hyperbolic equations, to 50% of JO_2max_ ([ADP] at 50% JO_2max_, variable with the same meaning of the apparent K_m_ for a MM kinetics) showed a trend to decrease after bed rest (*p* = 0.11) (see Figure 3, lower panel). The 43% decrease of the mean values of this variable, in POST versus PRE, suggests a greater sensitivity of mitochondrial respiration to submaximal [ADP] following bed rest.

**FIGURE 4:**
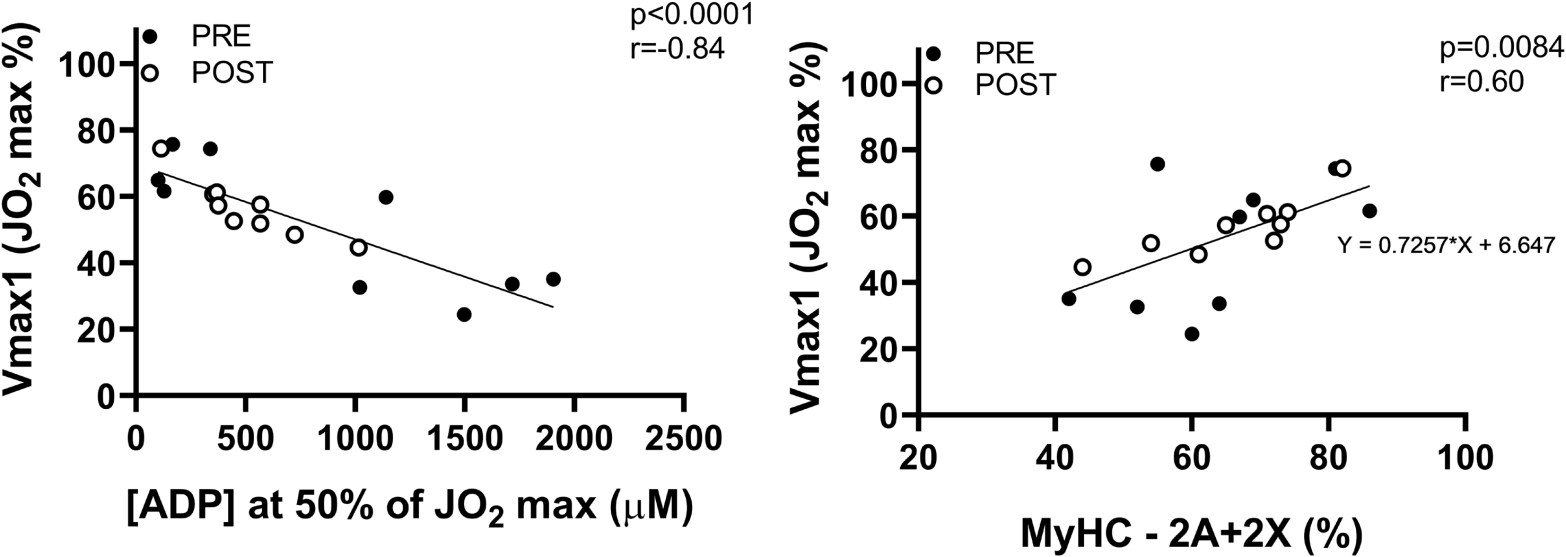
An inverse linear relation between V_max1_ and [ADP] values corresponding to 50% of maximal JO_2_ was observed (left panel). A positive linear relation between V_max1_ and the percentage of MyHC‐2A + 2X was also observed (right panel). Individual values obtained PRE and POST bed rest are shown. See text for further details.

Individual values of V_max1_ (sequential two‐hyperbolic approach) as a function of [ADP] at 50% JO_2max_ are shown in the left panel of Figure 4. An inverse and significant linear relationship was found between the variables. In the right panel of Figure 4, V_max1_ values are plotted as a function of the percentage of MyHC 2A + 2X. A positive and significant linear relationship was observed between the variables. Values of MyHC 1 and MyHC 2A + 2X isoforms expression did not change following bed rest [29].

**FIGURE 5:**
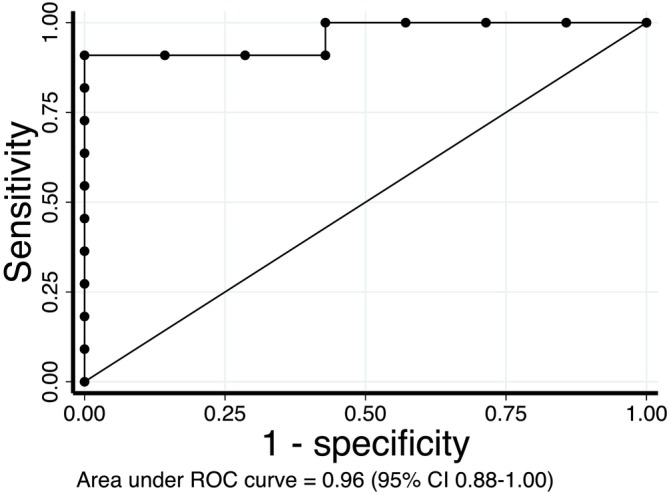
A receiver operating characteristic curve (ROC) analysis was conducted in order to investigate the predictive capacity by MyHC 2A + 2X to discriminate when the value of [ADP] at 50% of JO_2max_ occurred in the domain of the first sequential hyperbolic function. See text for further details.

A ROC curve analysis was conducted to investigate the predictive capacity by MyHC 2A + 2X to discriminate when the value of [ADP] at 50% of JO_2max_ occurred in the domain of the first sequential hyperbolic function. We further investigated whether there was a cut‐off value for the percentage of the less oxidative MyHC isoforms, which could predict a greater contribution by the first phase to the overall JO_2_ versus [ADP] response. A ROC curve lying on the diagonal line would indicate a low predictive capacity by MyHC 2A + 2X to discriminate when the value of [ADP] at 50% of JO_2max_ occurred in the domain of the first sequential hyperbolic function. The analysis demonstrated an excellent predictive performance by MyHC 2A + 2X, with an area under the ROC curve (AUC) of 0.96 (95% confidence interval 0.88–1.00) (see Figure 5). ROC analysis also identified a cut‐off value for MyHC 2A + 2X of 65%. Hence, for values of MyHC 2A + 2X greater than 65%, the value of [ADP] at 50% of JO_2max_ would reside in the domain of the first sequential hyperbolic function.

### 3.2 Data Obtained on Rat Muscle

**FIGURE 6:**
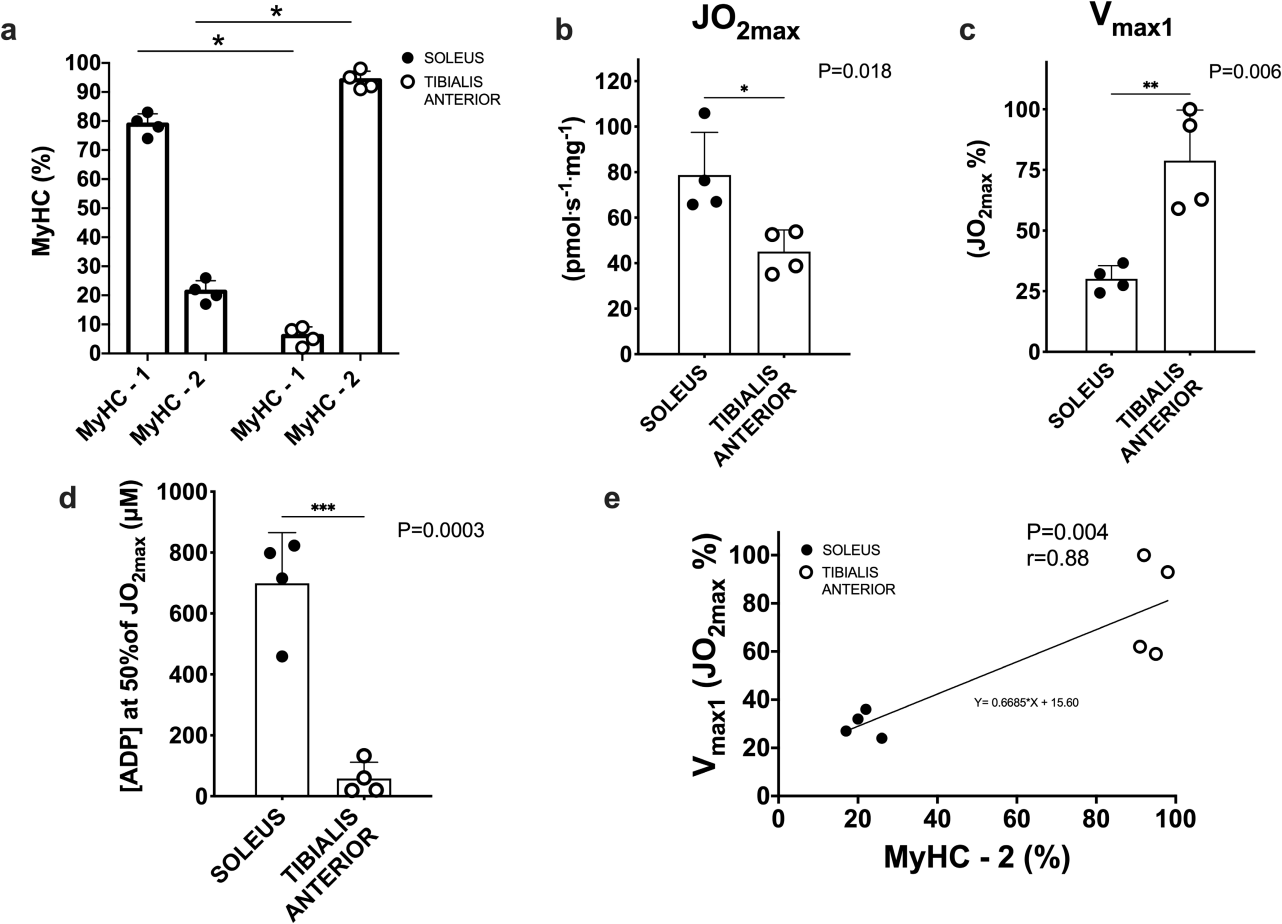
Data obtained in rat muscles. (a) Individual and mean (± SD) values of MyHC isoform percentages in soleus and tibialis anterior muscles. (b‐c) Individual and mean (± SD) values of maximal ADP‐stimulated mitochondrial respiration (JO_2max_) and V_max1_. (d) Individual and mean (± SD) values corresponding, for the sequential two‐hyperbolic equations, to the apparent K_m_ for a MM kinetics ([ADP] at 50% of JO_2max_) in soleus and tibialis anterior. (e) A positive linear relation between V_max1_ and the percentage of MyHC‐2A + 2X was also observed. Individual values are shown. See text for further details.

We further performed experiments on rat muscle (see Figure 6). Representative electrophoresis gels for the dosage of MyHCs percentage in the soleus and tibialis anterior are shown in Figure S4. As expected, the percentage of Type 1 fibres was higher and the percentage of Type 2 fibres was lower in the highly oxidative soleus versus the mixed muscle tibialis anterior (Panel A in Figure 6). Maximal ADP‐stimulated mitochondrial respiration was higher in the soleus versus the tibialis (Panel B in Figure 6). V_max1_ was higher in tibialis versus soleus (Panel C in Figure 6) and the [ADP] corresponding to 50% of JO_2max_ was lower in tibialis versus soleus (Panel D in Figure 6). Finally, V_max1_ data significantly correlated with the % of Type 2 fibres (Panel E in Figure 6). The slope of the correlation closely aligned with that observed in human experiments (Panel E in Figure 6).

## 4 Discussion

The main results of the present study can be summarized as follows: (i) In isolated permeabilized *vastus lateralis* fibres, two‐phase component models provided an alternative fit of the mitochondrial JO_2_ versus [ADP] data compared to the classic MM kinetics. (ii) When fitting the data with two sequential hyperbolic functions, two distinct phases of JO_2_ versus [ADP] became evident: A first phase was characterized by a lower maximal mitochondrial respiration (V_max1_) and a higher ADP sensitivity (lower apparent K_m1_) values, compared to V_max2_ and apparent K_m2_ determined for the second phase. (iii) The two sequential hyperbolic functions were solved, and the [ADP] value corresponding to 50% of JO_2max_ was calculated. This parameter showed a trend to decrease after bed rest, suggesting a greater sensitivity of mitochondrial respiration to submaximal [ADP]. (iv) Correlation and ROC analyses suggest that the two different phases in the mitochondrial respiration are related to the percentage of myosin heavy chain (MyHC) isoforms: More specifically, the first phase of the JO_2_ versus [ADP] response would be related to the percentage of less oxidative MyHC isoforms (Type 2A + 2X). (v) These conclusions were confirmed in control experiments carried out in male rat muscle samples with different percentages of MyHC isoforms and fibre types.

Fitting experimental data with higher order equations inevitably improves the quality of the fitting, as was the case in the present study for the two sequential hyperbolic functions compared to the single hyperbolic MM equation. In the present study, the *r* ^2^ increase between MM and the hyperbolic model (mean values from about 0.88 to about 0.96) was relatively small, although statistically very significant. From Figure 1 (data fitting and residual plots), it is evident that the MM equation overestimates the experimental data at low [ADP] and underestimates them at high [ADP].

More importantly, the MM equation did not allow to discriminate the two phases of the response, which represent the ‘core’ of the present study: Does the higher order fitting allow us to gain insights into physiological mechanisms? The data obtained in the present study strongly suggest that the answer is positive: The two sequential hyperbolic fitting allowed to distinguish two distinct phases of the JO_2_ versus [ADP] response. Each phase was characterized by different V_max_ and apparent K_m_ parameters, which were associated with a different composition of MyHC isoforms, thereby suggesting the presence of two distinct responses or contributions in the overall mitochondrial respiration.

In some previous studies, primarily conducted in animal models, data analysis did not follow the traditional MM fitting [20, 22, 23, 24, 25], already suggesting the presence of two different contributions in the submaximal mitochondrial respiration linked to fibre composition [20, 23, 24]. Saks et al. [20] proposed two superimposed hyperbolic equations, an approach which was utilized also in the present study (see Equation 2). The approach improved the fitting quality but could not distinguish the two phases. In our previous paper [1], we attempted a two‐phase fitting; however, the mathematical approach we utilized was directly derived from methods utilized in the analysis of O_2_ uptake kinetics [33] and could not yield information about physiologically relevant parameters, such as the apparent K_m_ and V_max_ of the different phases. These limitations were overcome in the present study.

The first phase which we identified was characterized by a high ADP sensitivity (low apparent K_m1_) and a low value of maximal respiration (V_max1_; see Figure 3). V_max1_ and K_m1_ were in the range of 11–42 pmol s^−1^ mg^−1^ and 10–221 μM, respectively. On the contrary, the second phase was characterized by a low ADP sensitivity (high apparent K_m2_) and a high value of maximal respiration (V_max2_). V_max2_ and K_m2_ were in the range of 27–87 pmol s^−1^ mg^−1^ and 408–3238 μM, respectively.

These data differ from those reported by Saks et al. [20]. The differences compared to some previous studies could be ascribed to factors related to the assay conditions and the substrate protocol. In the present study, we utilized blebbistatin, which prevents spontaneous contraction in the respiration medium by inhibiting myosin II‐ATPase [28]. Moreover, the measurements were conducted at a physiological temperature of 37°C instead of 25°C [20, 23]. In terms of the substrate protocol, the submaximal ADP titrations were performed in the presence of succinate, which generates superoxide during reverse electron transport into complex I [34]. This may affect K_m_ due to ROS‐dependent modifications of redox‐sensitive components of the phosphorylation systems. Likewise, ROS‐independent differences between substrate designs might affect K_m_ values once ADP is titrated. Most of the previous studies were conducted in the absence of succinate and in the presence of glutamate/pyruvate and malate [20, 27]. Preliminary data from our lab provide further support for the presence of a two‐phase component model, even under conditions that do not involve complex II activation by succinate (see Figure S5).

We hypothesized that the two phases in the JO_2_ versus [ADP] response, well documented by the sequential two‐hyperbolic functions, might be related to fibre types with different MyHC compositions. The human *vastus lateralis* muscle is typically a mixed muscle. Values of MyHC 1 and MyHC 2A + 2X isoforms expression, measured via gel electrophoresis in the subjects of the present study were 35% ± 18% and 65% ± 12%, respectively, with no significant changes following the 10‐day bed rest [29]. More specifically, we hypothesized that the first phase of the JO_2_ versus [ADP] kinetics would be related to less oxidative (Type 2A + 2X) MyHC isoforms, characterized by low apparent K_m1_ and V_max1_ values. On the other hand, the second phase of the JO_2_ versus [ADP] kinetics would relate to more oxidative (Type 1) MyHC isoforms, characterized by high apparent K_m2_ and V_max2_ values.

The hypothesis was supported by the experimental results. We indeed observed a significant correlation between individual V_max1_ values and the percentage of MyHC 2A + 2X isoforms protein expression (Figure 4). In other words, higher V_max1_ correlated with a higher percentage of less oxidative MyHC isoforms. Although a correlation between two variables does not imply cause ‐ effect, it does support the hypotheses mentioned above.

Further support for the concept of a relation between the first phase of the JO_2_ versus [ADP] response and Type 2A + 2X MyHC isoform derives from ROC analysis presented in Figure 5. In this case, the question we posed was the following: Does the percentage of less oxidative MyHC isoforms predict if the [ADP] value corresponding to 50% of JO_2max_ will occur in the first phase of the JO_2_ versus [ADP] kinetics? And, moreover, is there a cut‐off value of the percentage of less oxidative MyHC isoforms that can predict whether the greater contribution to the overall JO_2_ versus [ADP] response is given by the first phase of the kinetics? The ROC curve and the calculated area under the curve (AUC 0.96; 95% confidence interval 0.88–1.00) showed that the percentage of less oxidative MyHC isoforms has a very strong predictive capacity to discriminate if the [ADP] value corresponding to 50% of JO_2max_ will occur in the first phase of the kinetics. Moreover, the analysis identified a cut‐off value (65%) of the percentage of Type 2A + 2X MyHC isoforms above which the greater contribution to the overall response is given by the first phase of the kinetics. In other words, if at least 65% of Type 2A + 2X MyHC isoforms are present, we can predict that the [ADP] value corresponding to 50% of JO_2max_ will occur in the first phase of the kinetics.

Whereas the relationship between mitochondria associated with less oxidative Type 2A + 2X MyHC isoforms and low maximal respiration (V_max1_) values or those associated with oxidative Type 1 MyHC isoforms and high maximal respiration (V_max2_) values appears straightforward (see also [25]), a lower sensitivity to submaximal [ADP] (higher apparent K_m2_) for mitochondria associated with oxidative Type 1 MyHC isoforms may appear counterintuitive. Given that mitochondrial volume varies across different fibre types [35], the differences in V_max_ may be related to mitochondrial content and CS activity. However, these differences do not seem to be fully eliminated even after normalization to CS activity [25, 35, 36]. Research on single fibres also indicates a distinct regulation of mitochondrial activity across fibre types, with mitochondria in more oxidative muscles exhibiting a lower affinity for ADP [24, 25, 37]. Also in studies carried out (as the present one) in permeabilized muscle fibres demonstrated that oxidative tissues, such as the heart or the soleus muscle, have very high values of the apparent K_m_ for ADP (200–500 μM), exceeding by far that of fibres with low oxidative activity (10–15 μM) [20, 25]. This would allow Type 1 muscle fibres to modulate oxidative phosphorylation with low ADP fluctuations, reducing the activation of glycolysis and glycogenolysis, and would also allow the fibres to operate at higher ADP concentrations, reducing the proton motive force and mitochondrial ROS formation [21, 38]. Fast‐twitch skeletal muscle, such as white, red and mixed gastrocnemius, showed a high affinity for ADP, with apparent K_m_ values for ADP (10–15 μM) similar to those found in isolated mitochondria.

More specifically, the low ADP sensitivity observed in Type 1 skeletal muscle might be due to a lower permeability of the outer mitochondrial membrane for ADP [20]. The outer mitochondrial membrane would restrict free ADP diffusion from the extramitochondrial space by maintaining the voltage‐dependent anion channel (VDAC) in a low‐conductance state [37, 39]. In addition, Type 1 muscle fibres seem to be more sensitive to creatine (Cr) concentration than Type 2 muscle fibres. In Type 1 muscle fibres cytosolic ADP would no longer be the main stimulus of mitochondrial respiration, which would be mainly driven by the local Cr‐to‐PCr (phosphocreatine) ratio, with mitochondrial creatine kinase being coupled to ATP production. This functional coupling seems not to be present in Type 2 muscle fibres [40]. The proposed muscle energetics model offers several advantages, particularly for Type 1 muscle fibres. It enables the modulation of oxidative phosphorylation with minimal ADP fluctuations, thereby limiting the activation of glycolysis and glycogenolysis. This mechanism also allows the fibres to operate at higher ADP concentrations, which in turn reduces the proton motive force and mitigates mitochondrial ROS production [21, 38].

The different kinetics may also result from the contributions of different mitochondrial subpopulations such as the intermyofibrillar (IMF) and subsarcolemmal mitochondria (SSM), as SSM seem to be more susceptible to changes in mitochondrial volume density with training/detraining [41].

A trend towards increased ADP sensitivity was observed after 10‐day bed rest. Although statistical significance was not reached (see Figure 3; *p* = 0.11), the [ADP] corresponding to 50% of JO_2max_ decreased by 43% following bed rest, suggesting a higher sensitivity of mitochondrial respiration to submaximal [ADP]. An increased ADP sensitivity following bed rest would be in accordance with previous findings in the literature, showing a decreased ADP sensitivity (higher apparent K_m_) of skeletal muscle fibres following training [42] and an increased ADP sensitivity (lower apparent K_m_) following a bed rest period of similar duration compared to that of the present study [10].

An impairment of the ‘transcriptome’ of mitochondrial genes occurs as early as after 5 days of bed rest [43]. Monti et al. [29] reported no changes in the protein expression of MyHC isoforms following 10 days of bed rest, but they observed an upregulation of MYH1 (encoding for MyHC‐2X) and a downregulation of MYH7 (encoding for MyHC‐1) detected by RNA sequencing. This discrepancy could be related to the fact that MyHC have a longer turnover time compared to their transcripts. The present finding of a trend towards an increased ADP sensitivity, together with the decreased K_m2_ values, may point towards an early stage of low‐to‐fast fibre transition, a well‐known phenomenon associated with inactivity and disuse, which after 10 days of bed rest would not yet occur at the level of protein expression but is already present at the transcriptomic level.

The link between the two‐phase model fitting and fibre type composition was confirmed by the control experiments carried out on rat skeletal muscle (see Figure 6). As expected, soleus and tibialis anterior muscles were characterized by distinct fibre type composition (Panel A in Figure 6) and different maximal ADP‐stimulated mitochondrial respiration values (Panel B in Figure 6). Confirming the data of the present study, V_max1_ was higher in less oxidative tibialis versus the more oxidative soleus (Panel C in Figure 6) and the lower values of [ADP] at 50% JO_2max_ in tibialis (versus soleus) demonstrate a greater sensitivity to submaximal [ADP] (Panel D in Figure 6). Finally, confirming the data of the present study, V_max1_ significantly correlated with the percentage of Type 2 fibres (Panel E in Figure 6).

A possible limitation of this study is that the high‐resolution respirometry experiments were conducted at 37°C, which may not fully reflect the local conditions of mitochondria in vivo [44]. Certain components of the mitochondrial electron transport chain may operate at higher local temperatures (~50°C) [45]. This temperature discrepancy could impact the interpretation of ex vivo data collected at the standard temperature of 37°C. A retrospective correction of our data, in order to take into account higher temperatures, seems however unfeasible, also considering that our measurements involved submaximal and maximal metabolic rates. A temperature correction would also make comparisons with previous studies impossible. Work conducted on intact cells and isolated liver mitochondria has shown that incubation at temperatures above 43°C can damage mitochondrial structure and function [46]. Future research could focus on these points and could investigate local temperature in different muscle fibre types and at different metabolic rates. Moreover, in the present study, we did not investigate the intracellular distribution, mitochondrial dynamics or potential variations in the mitochondrial network or microenvironment, which could also contribute to the biphasic kinetics observed. These factors might influence mitochondrial function and further explain the observed differences in respiratory behaviour between muscle fibre types. Moreover, whereas blebbistatin was used to limit spontaneous contraction, we recognize that its translational relevance to human physiology is debated, as prior studies [47] suggest its presence may not impact oxygen flux when evaluating OXPHOS capacity in human fibre bundles. In addition, blebbistatin is known to be photosensitive and specifically inactivated by blue light with wavelengths below 500 nm [48], and conducting experiments with the chamber lights turned off may be advisable to minimize potential photodegradation effects. However, considering that the light source of the oxygraph operates predominantly in the orange spectrum (wavelengths above 500 nm), it may not significantly influence blebbistatin's activity.

In conclusion, in the present study, we utilized, for the analysis of mitochondrial respiration sensitivity to submaximal [ADP], a novel mathematical approach (two sequential hyperbolic functions) for the fitting of JO_2_ versus [ADP] data obtained by high‐resolution respirometry on permeabilized skeletal muscle fibres obtained from subjects exposed to a 10‐day bed rest. Our novel approach provided an alternative fitting of the experimental data compared to the traditional MM kinetics equation, and allowed to identify two distinct phases of the response, related to fibre type composition. A first phase, characterized by low apparent K_m_ and V_max_ values, was correlated with the percentage of less oxidative (Type 2A + 2X) MyHC isoforms. A second phase, characterized by high apparent K_m_ and V_max_, was related to more oxidative (Type 1) MyHC isoforms.

## Ethics Statement

All human and animal studies have been approved by the appropriate ethics committee and have therefore been performed in accordance with the ethical standards laid down in the 1964 Declaration of Helsinki and its later amendments. All persons gave their informed consent prior to their inclusion in the study.

## Conflicts of Interest

The authors declare no conflicts of interest.

## Supporting information


**Figure S1** Representative traces of ADP‐stimulated mitochondrial respiration in permeabilized skeletal muscle fibres from the *vastus lateralis* in humans (A) and the *soleus* (B) and *tibialis* (C) muscles in rats. The blue line represents the oxygen concentration ([μM]), while the red line shows the mass‐specific O₂ flux normalized to mg of wet weight of skeletal muscle over time. Vertical line markers indicate the addition of specific substrates and chemicals used in the protocol: ‘blebb’ denotes the addition of blebbistatin; ‘G + M’ represents glutamate and malate; ‘S’ is succinate; ‘ADP’ indicates submaximal ADP concentrations; and ‘CIT C’ is cytochrome c. See text for further details.

**Figure S2** Individual oxygraph traces (*n* = 52) of ADP‐stimulated mitochondrial respiration in permeabilized skeletal muscle fibres from the *vastus lateralis* in humans and in the *soleus* and *tibialis* muscles in rats. See text for further details.

**Figure S3** *ADP‐stimulated mitochondrial respiration.* In the upper panels, respiration rates (JO_2_, absolute values) as a function of [ADP] in a typical subject PRE bed rest are shown. Data were fitted using three different mathematical models. K_m_ indicates the [ADP] at 50% of JO_2max_; K_m1_ and K_m2_ are the [ADP] values needed to stimulate the 50% of V_max1_ and V_max2_, respectively. In the lower panels, analysis of residuals showed an increased quality of the fitting for the superimposed and for the sequential two‐hyperbolic functions compared to the traditional MM kinetics equation.

**Figure S4** Coomassie‐stained electrophoresis gels showing the content of MyHCs in the four rats’ soleus and tibialis anterior muscles.

**Figure S5** Graphs from one wild‐type mouse (male, 18 months old, *tibialis muscle*) and one human “Subject 1” (male, age 77 years old, biopsies obtained from *vastus lateralis*), in which ADP sensitivity was evaluated in the presence of blebbistatin (25 μM), glutamate (10 mM) and malate (4 mM). The two‐phase kinetics were observed even when succinate was not present.
