# Supplementary figures and images for: Mitochondrial Sensitivity to Submaximal [ADP] Following Bed Rest: A Novel Two‐Phase Approach Associated With Fibre Types
Source: J Cachexia Sarcopenia Muscle. 2025 Apr 25;16(3):e13775. doi: 10.1002/jcsm.13775 (PMC12031883; doi:10.1002/jcsm.13775)

HUMANS

A

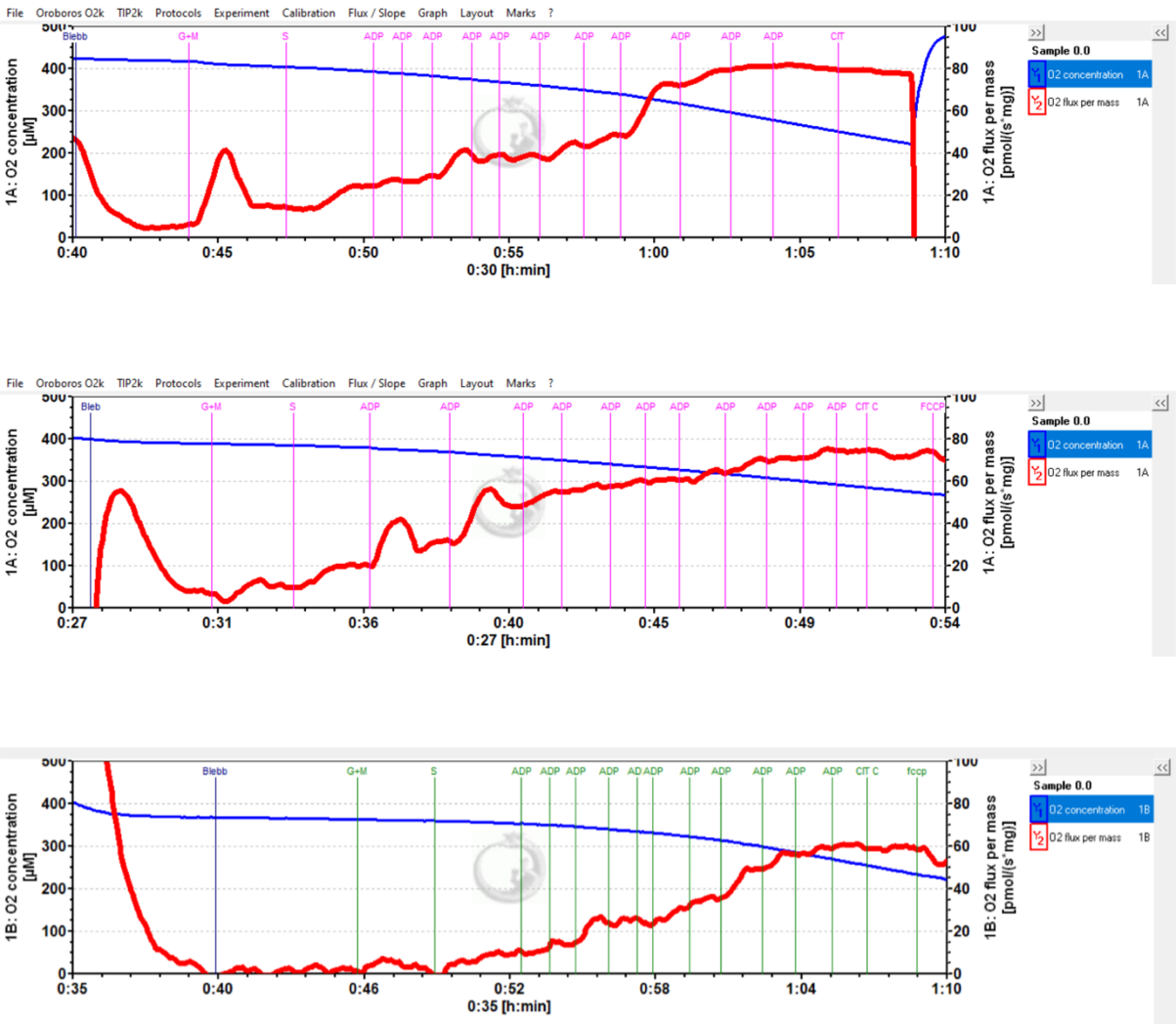

## B

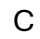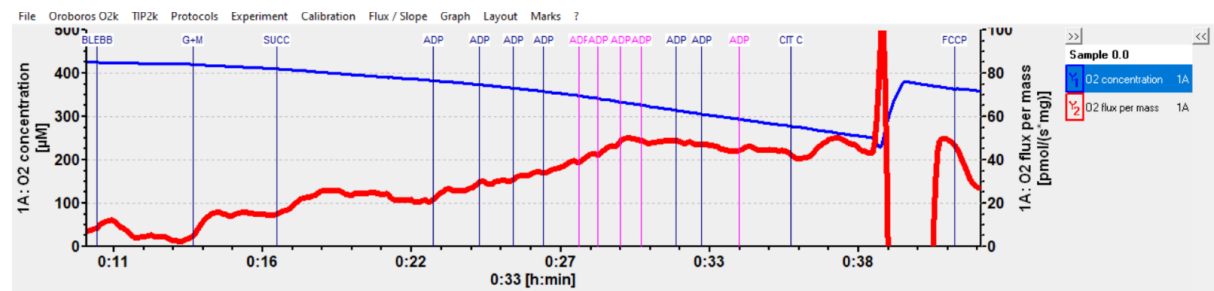

Supplement: Supplementary file 1 — Figure S1 Representative traces of ADP‐stimulated mitochondrial respiration in permeabilized skeletal muscle fibres from the vastus lateralis in humans (A) and the soleus (B) and tibialis (C) muscles in rats. The blue line represents the oxygen concentration ([μM]), while the red line shows the mass‐specific O₂ flux normalized to mg of wet weight of skeletal muscle over time. Vertical line markers indicate the addition of specific substrates and chemicals used in the protocol: ‘blebb’ denotes the addition of blebbistatin; ‘G + M’ represents glutamate and malate; ‘S’ is succinate; ‘ADP’ indicates submaximal ADP concentrations; and ‘CIT C’ is cytochrome c. See text for further details. [file JCSM-16-e13775-s005.pdf]

## HUMANS

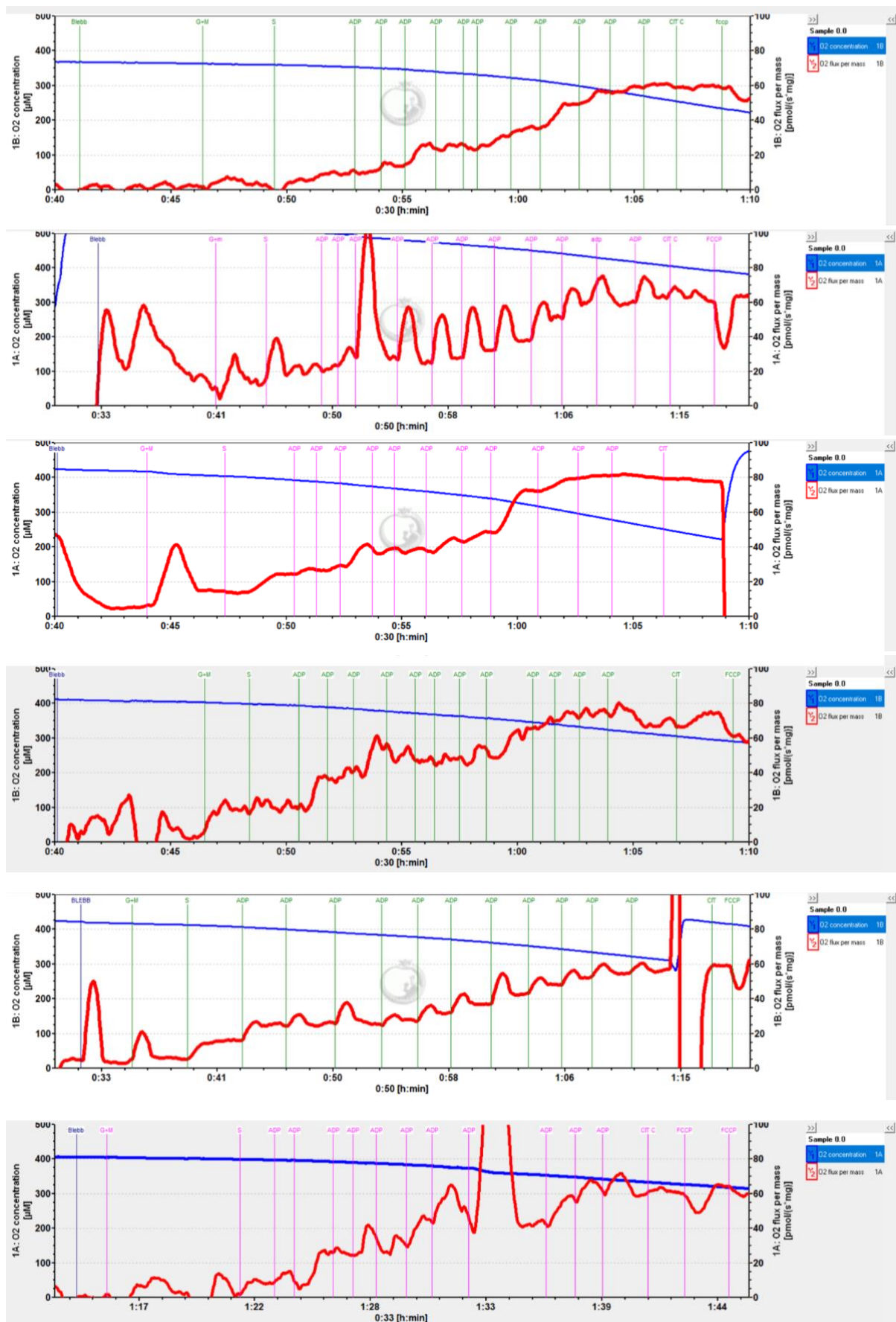

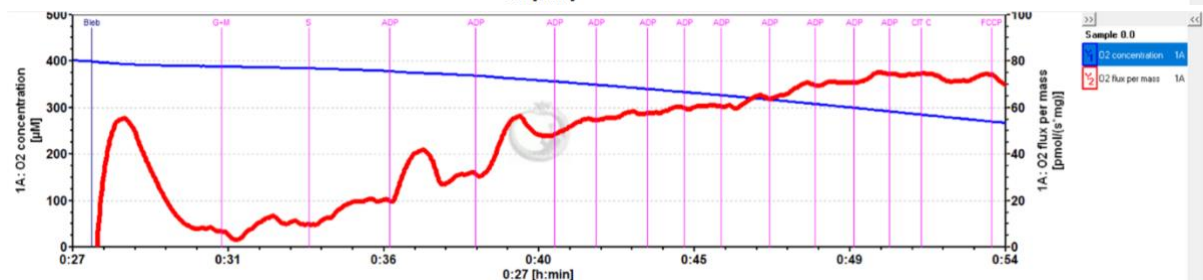

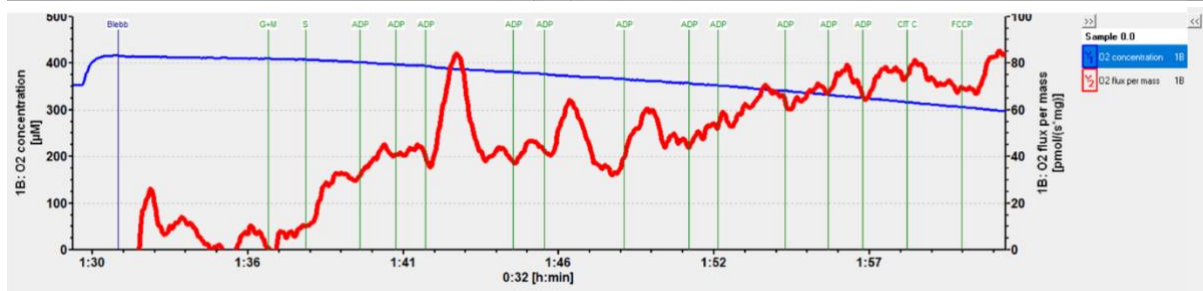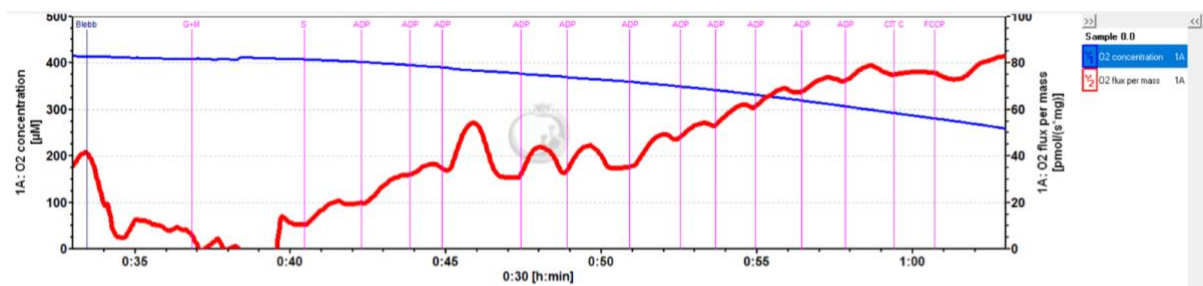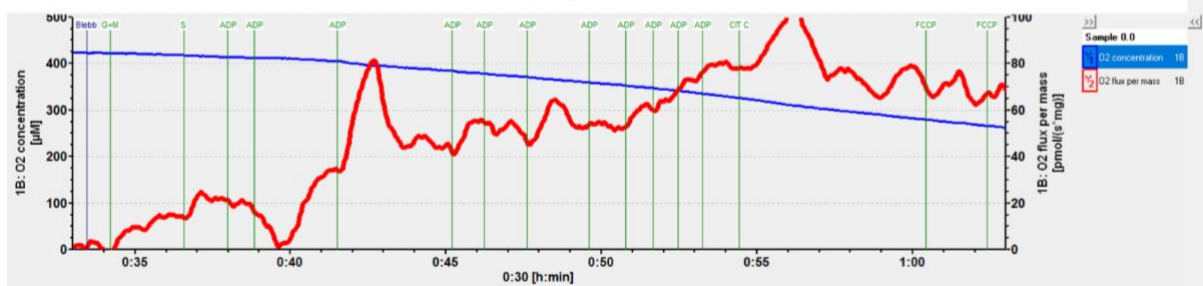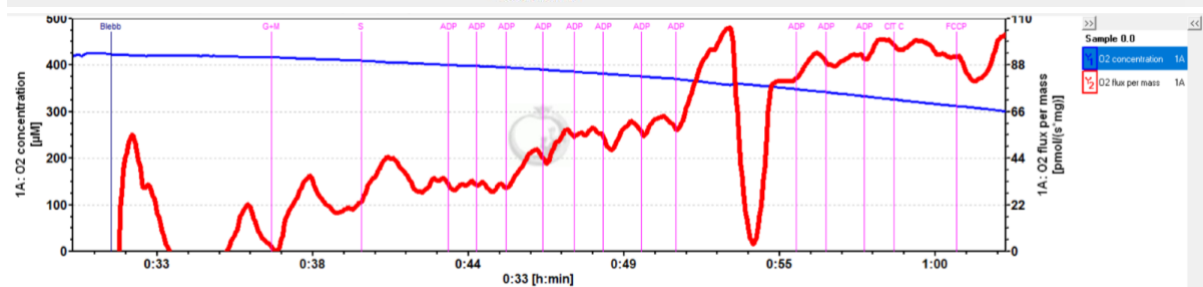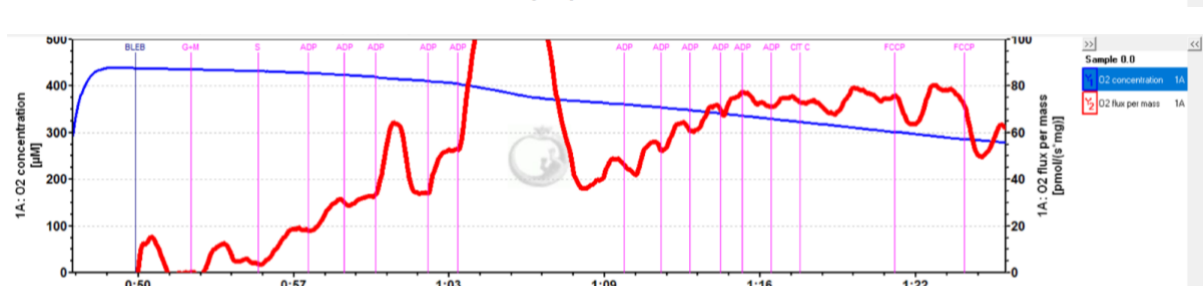

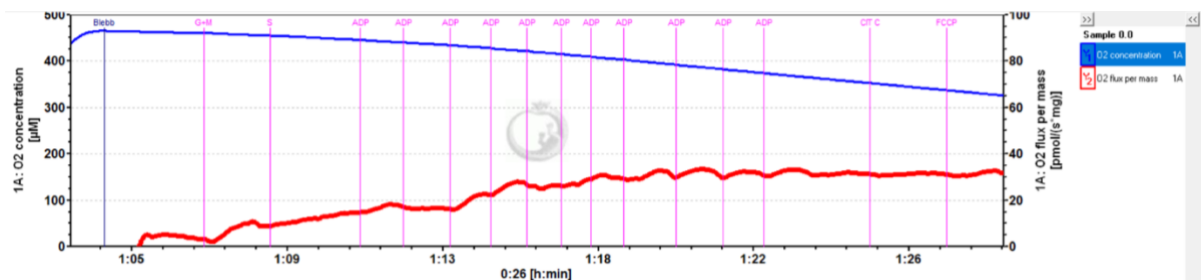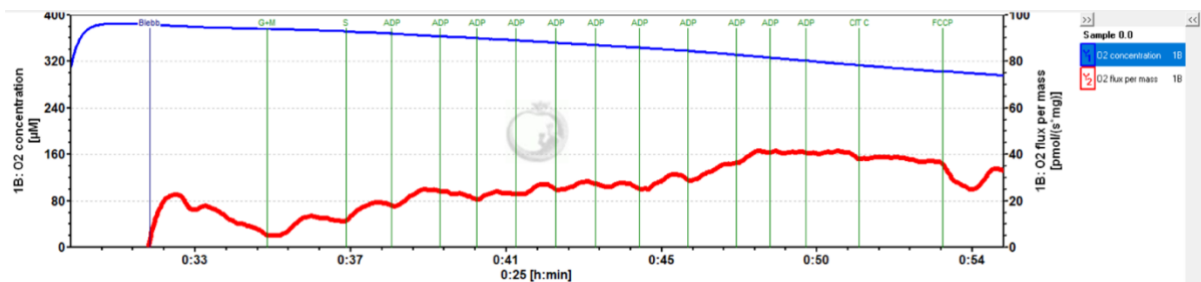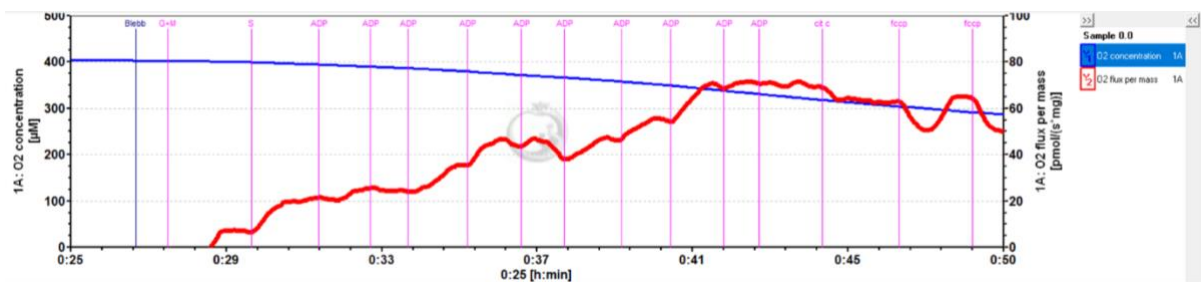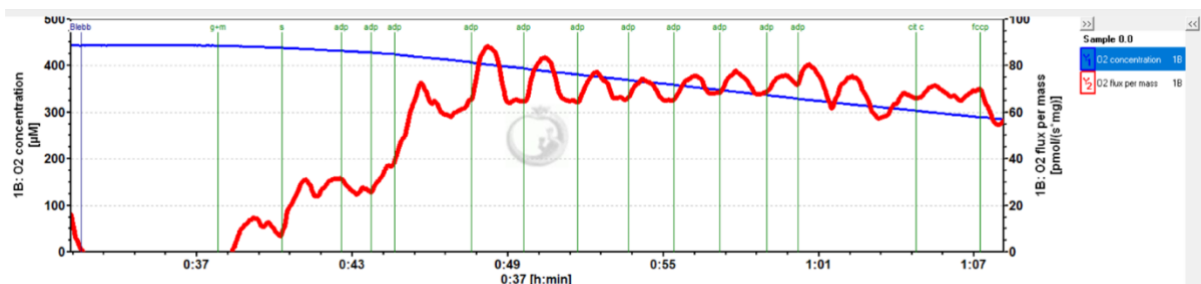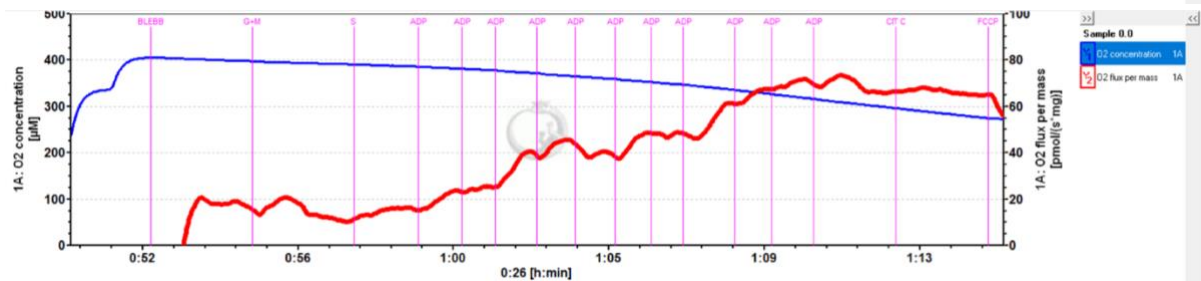

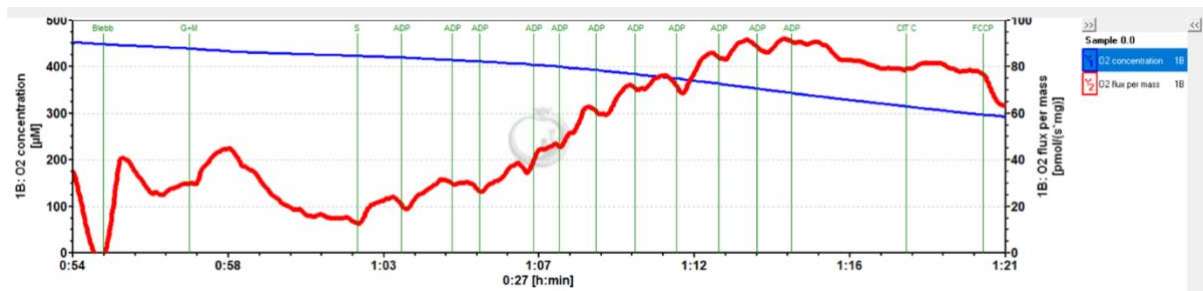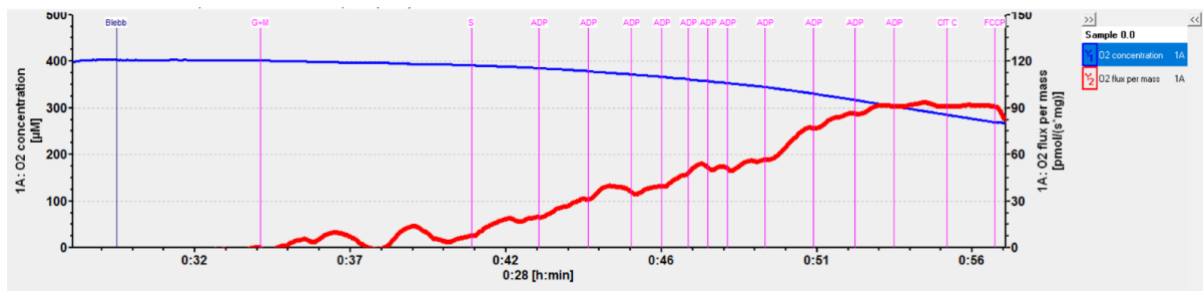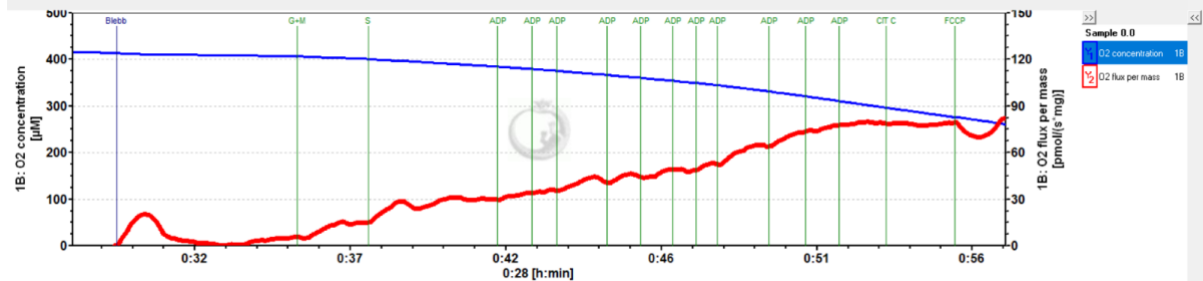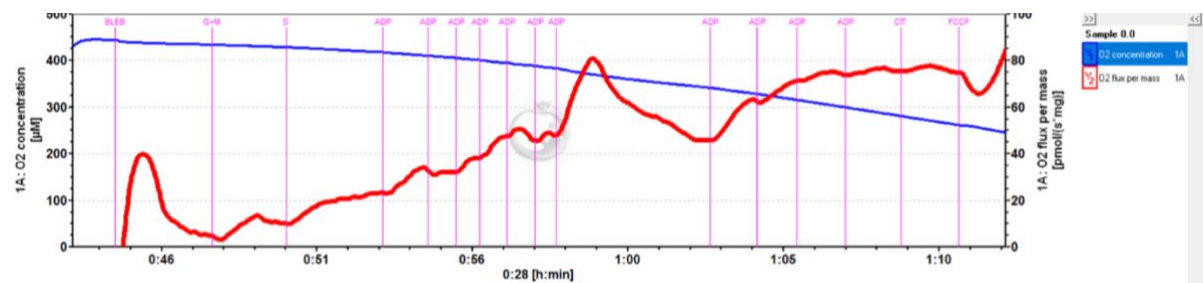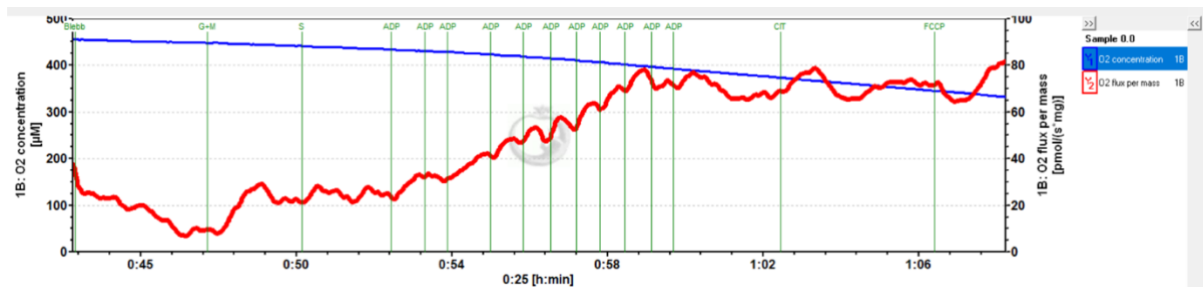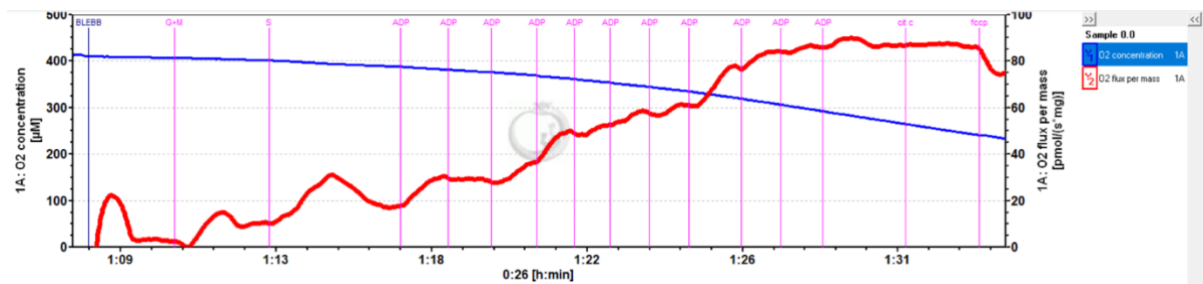

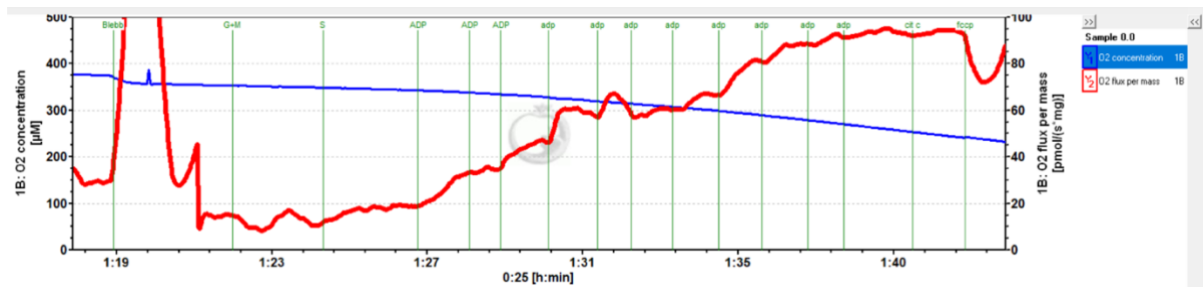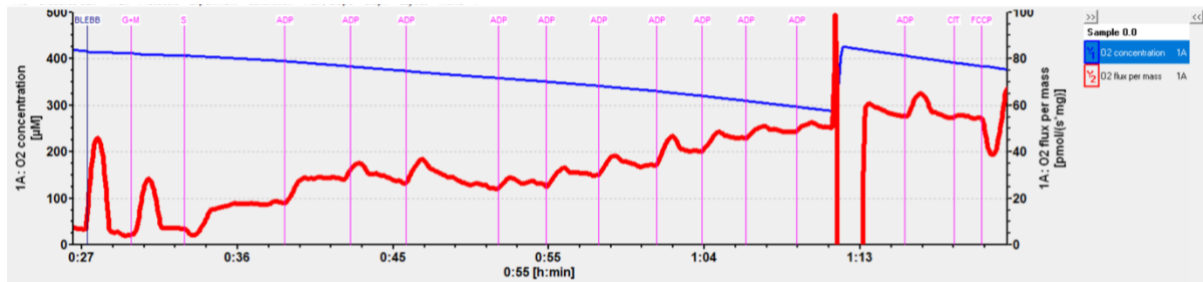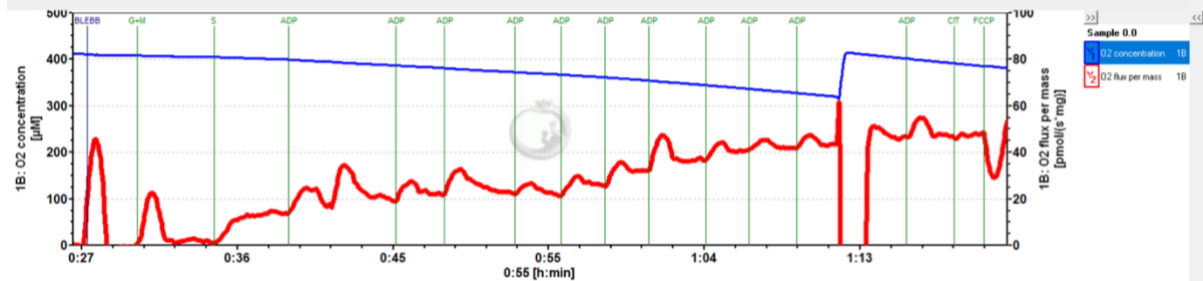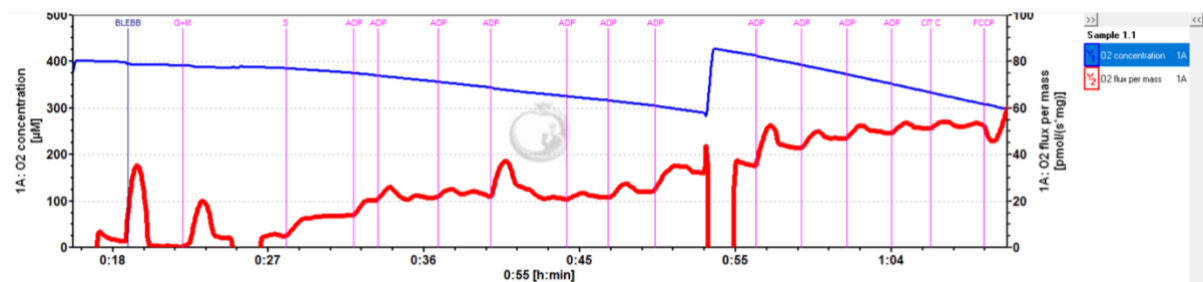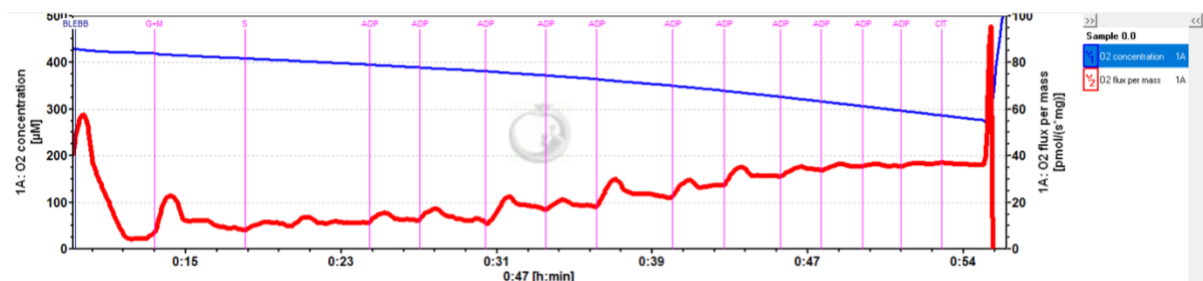

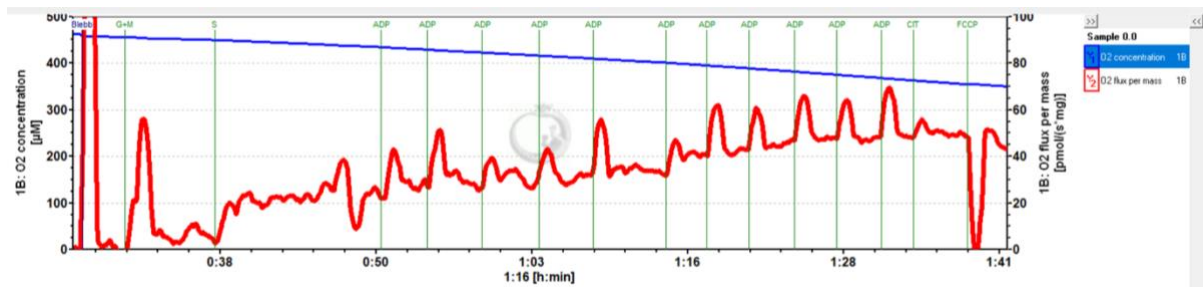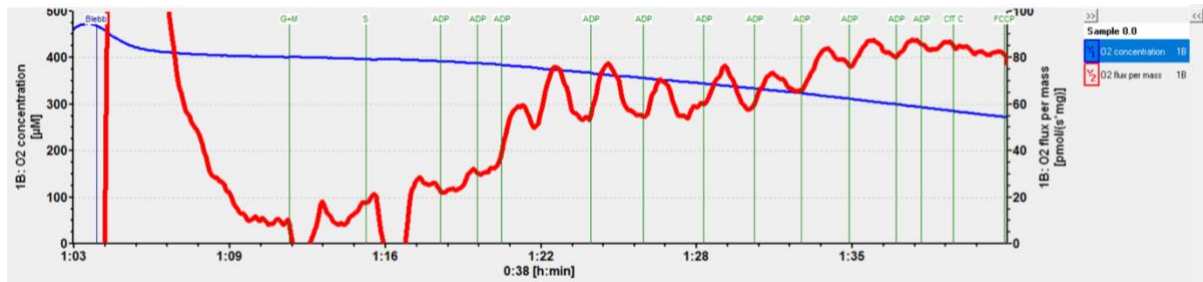

[illegible]

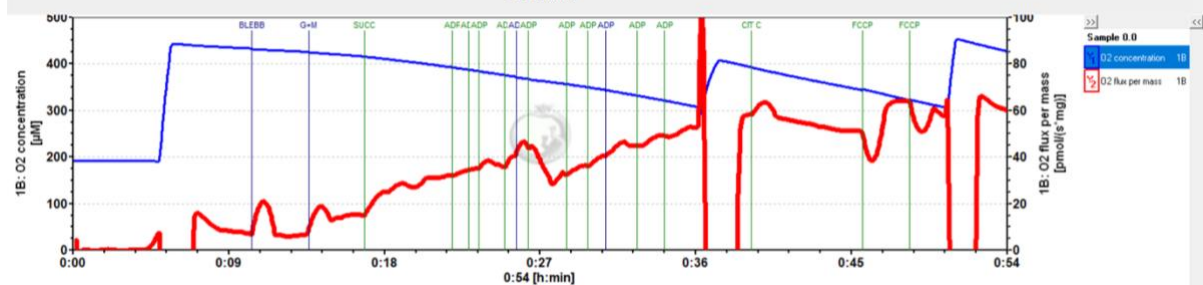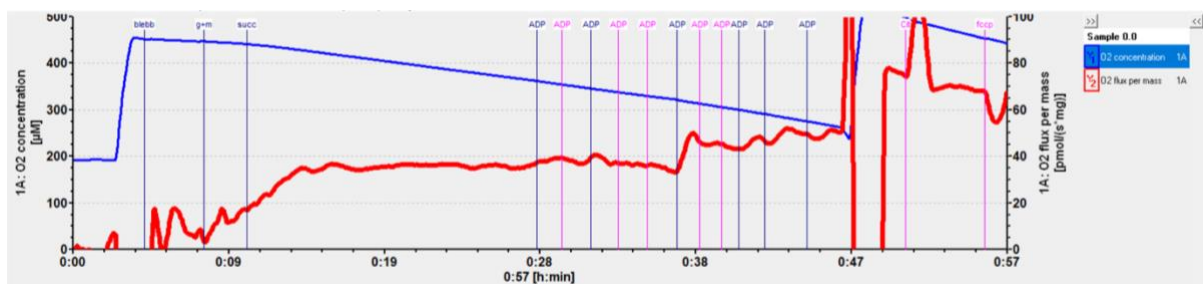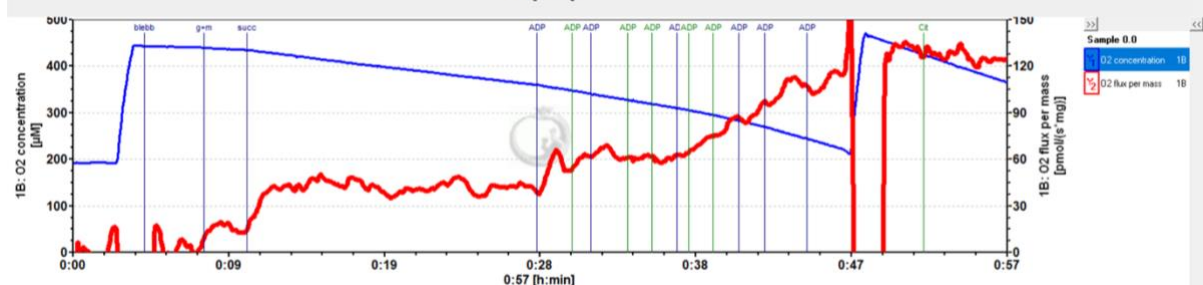

## TIBIALIS

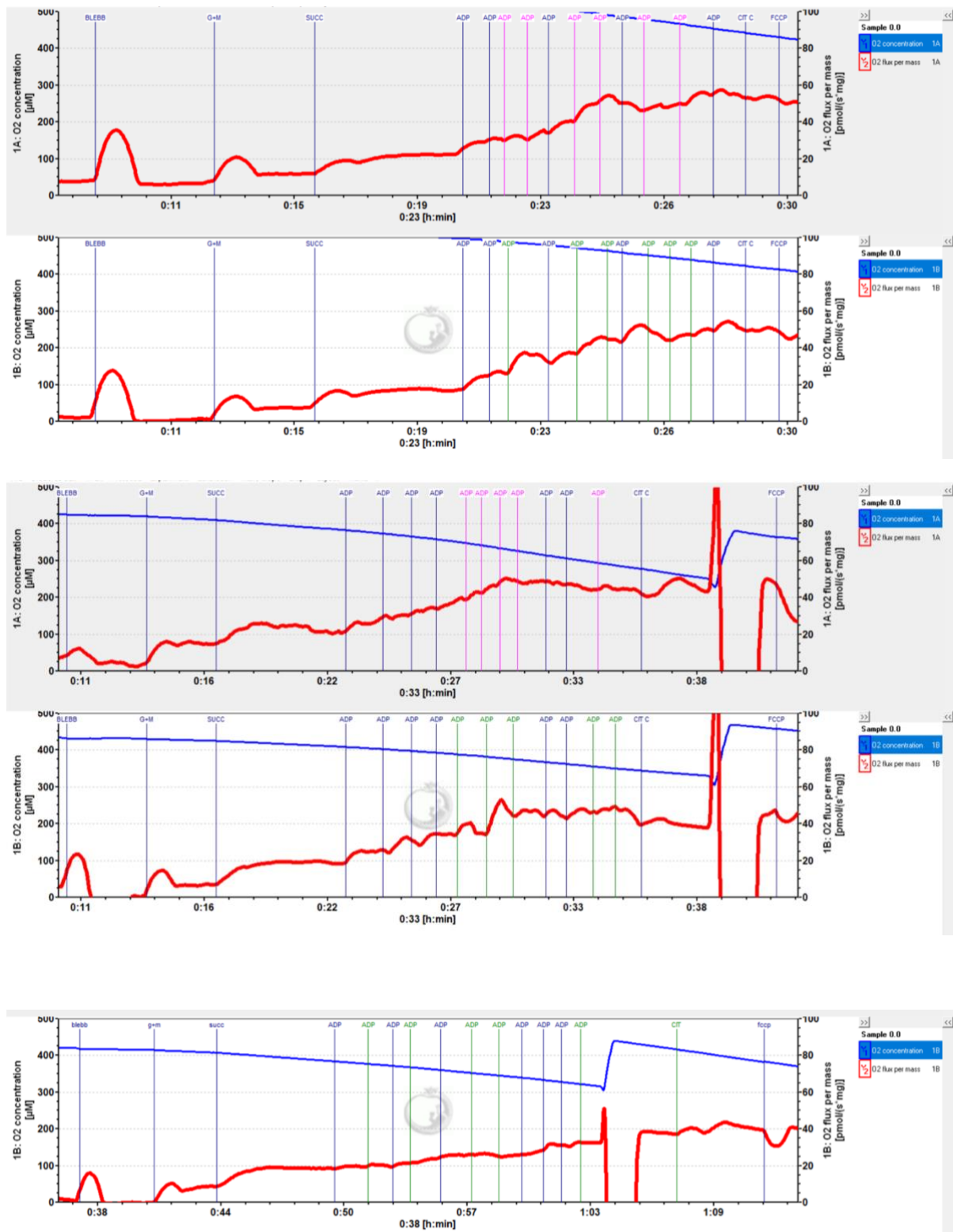

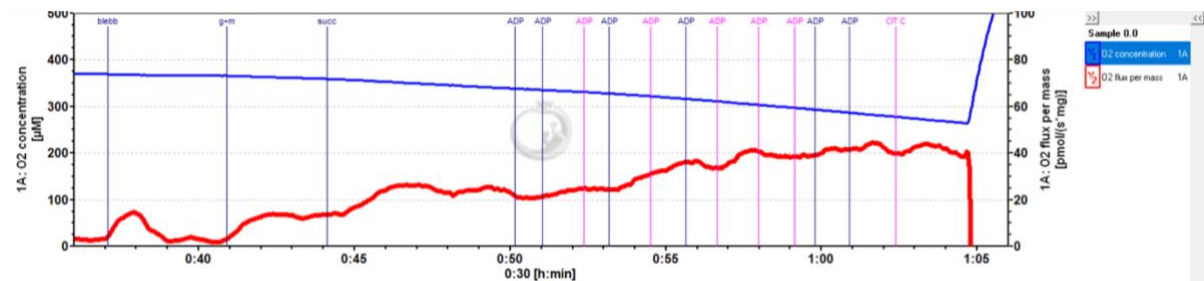

Supplement: Supplementary file 2 — Figure S2 Individual oxygraph traces (n = 52) of ADP‐stimulated mitochondrial respiration in permeabilized skeletal muscle fibres from the vastus lateralis in humans and in the soleus and tibialis muscles in rats. See text for further details. [file JCSM-16-e13775-s001.pdf]

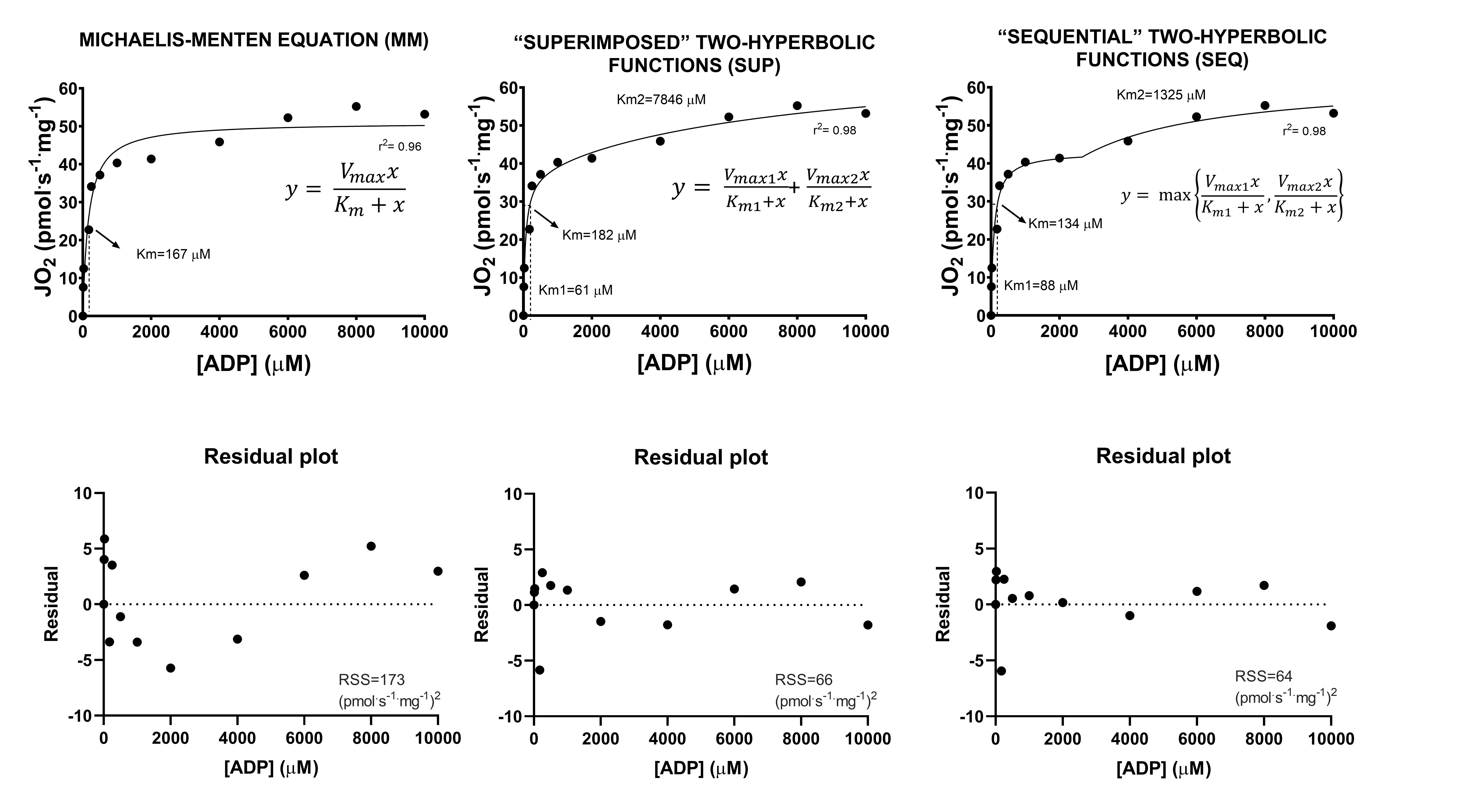

Supplement: Supplementary file 3 — Figure S3 ADP‐stimulated mitochondrial respiration. In the upper panels, respiration rates (JO2, absolute values) as a function of [ADP] in a typical subject PRE bed rest are shown. Data were fitted using three different mathematical models. Km indicates the [ADP] at 50% of JO2max; Km1 and Km2 are the [ADP] values needed to stimulate the 50% of Vmax1 and Vmax2, respectively. In the lower panels, analysis of residuals showed an increased quality of the fitting for the superimposed and for the sequential two‐hyperbolic functions compared to the traditional MM kinetics equation. [file JCSM-16-e13775-s003.tif]

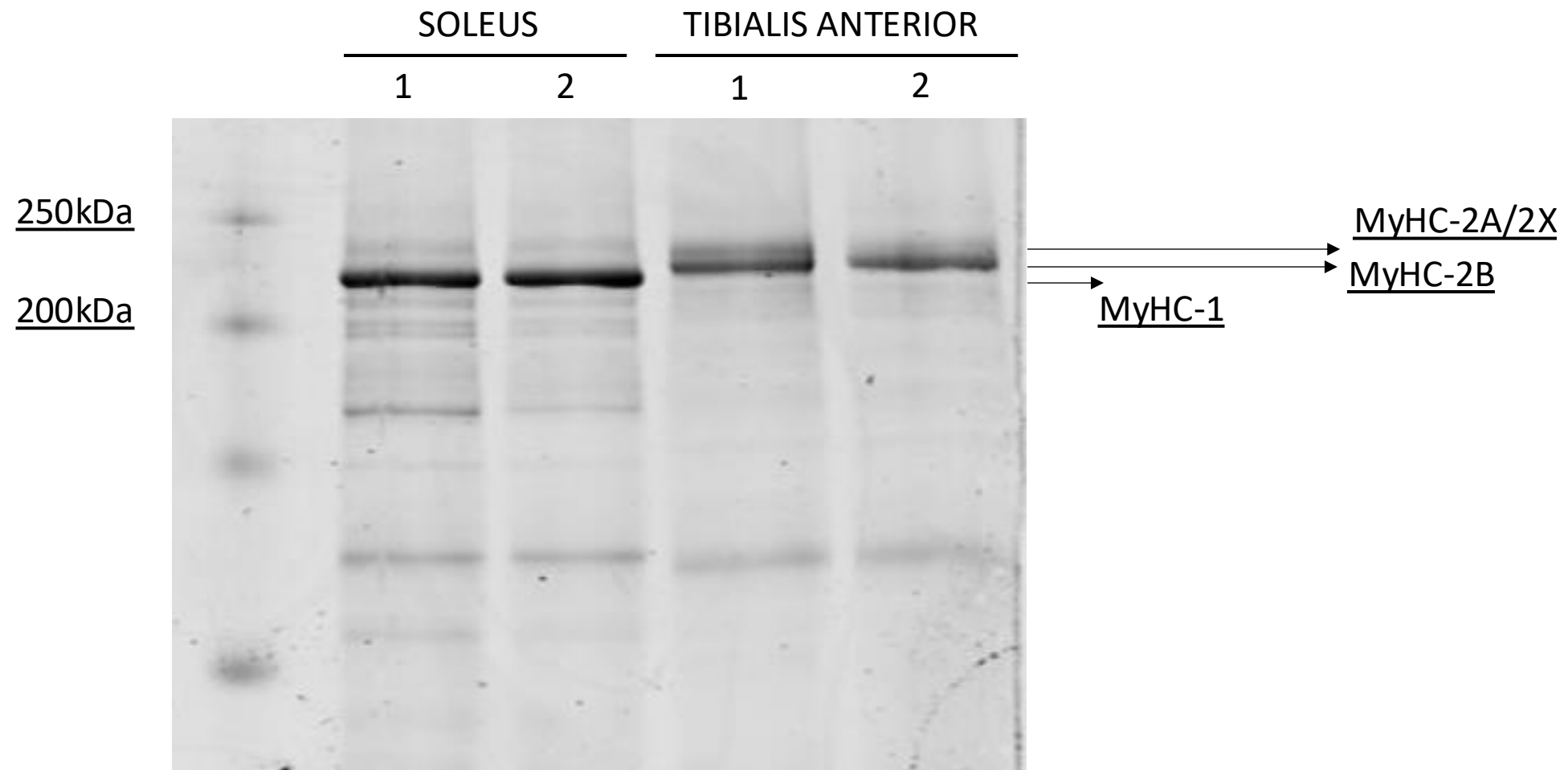

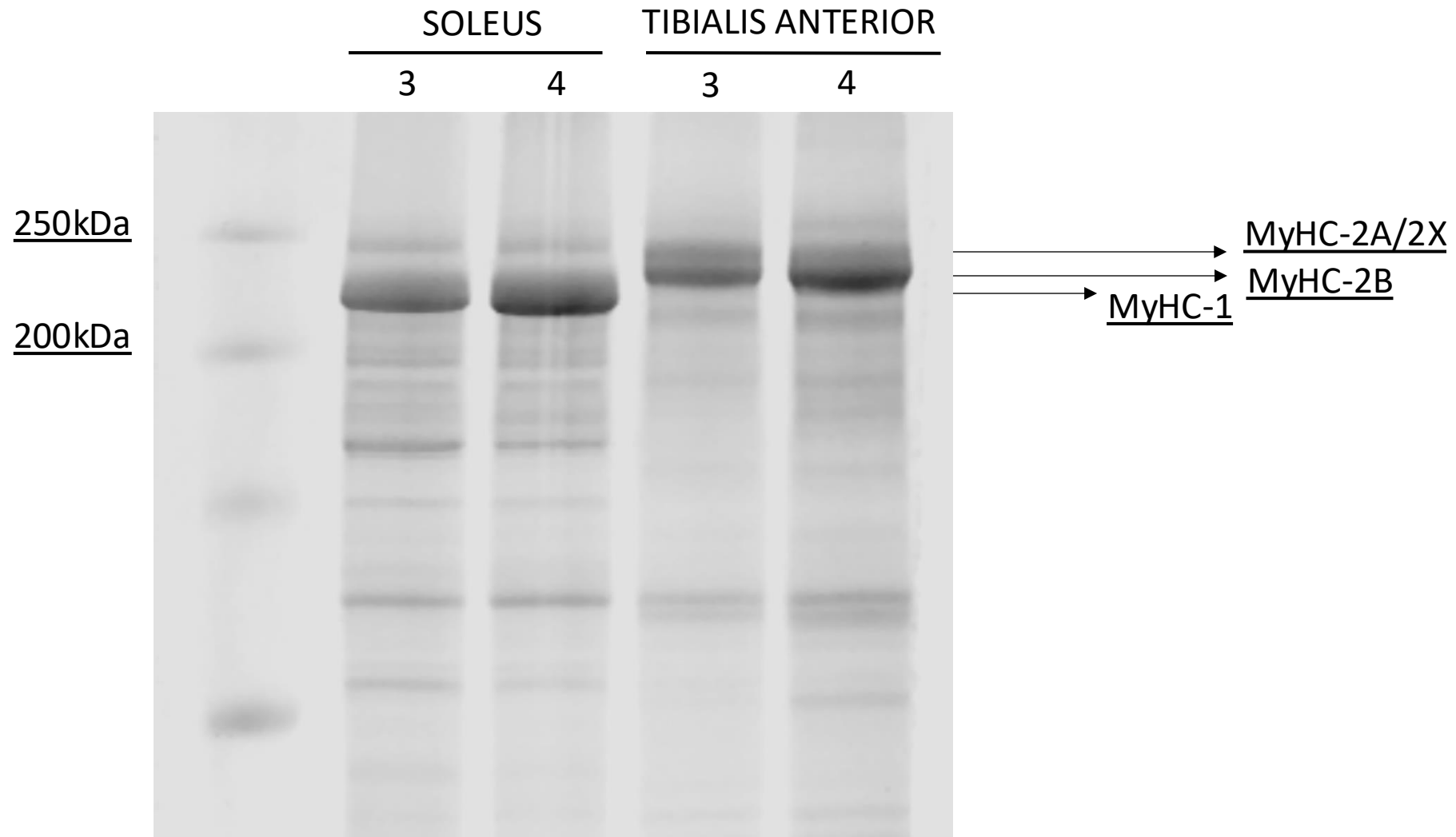

Supplement: Supplementary file 4 — Figure S4 Coomassie‐stained electrophoresis gels showing the content of MyHCs in the four rats’ soleus and tibialis anterior muscles. [file JCSM-16-e13775-s004.pdf]

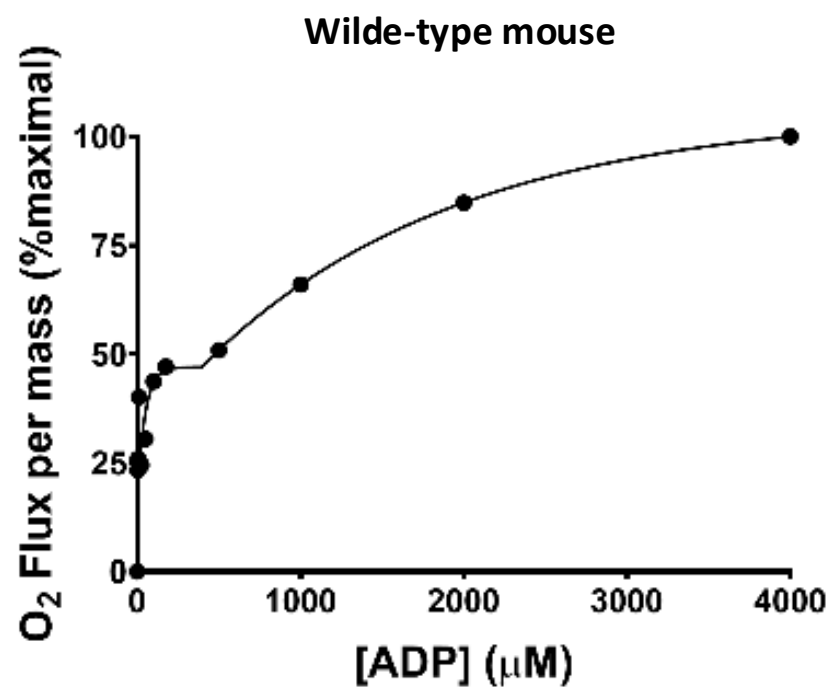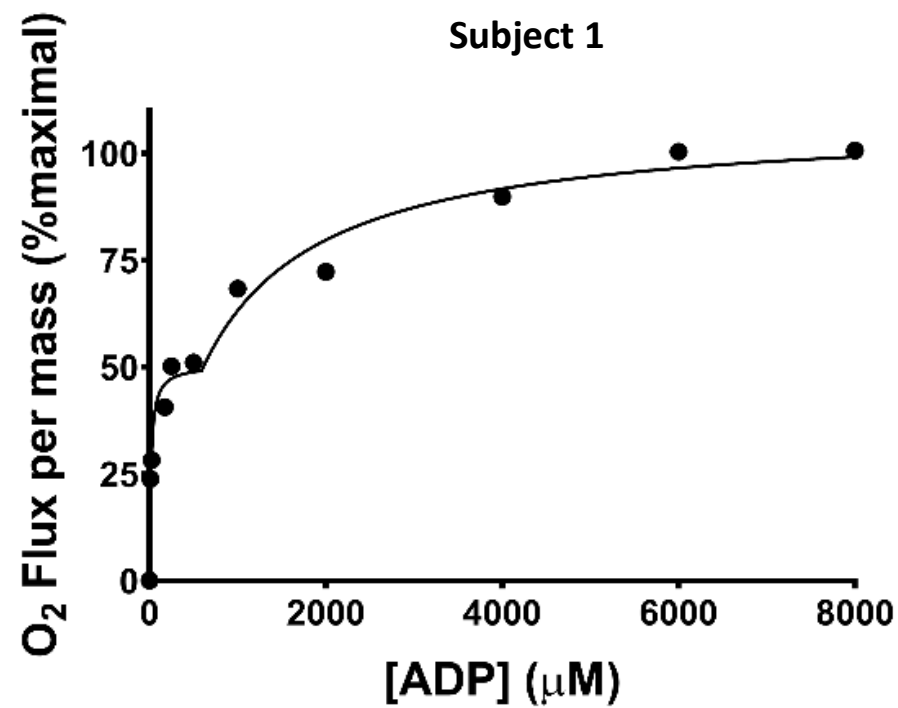

Supplement: Supplementary file 5 — Figure S5 Graphs from one wild‐type mouse (male, 18 months old, tibialis muscle) and one human “Subject 1” (male, age 77 years old, biopsies obtained from vastus lateralis), in which ADP sensitivity was evaluated in the presence of blebbistatin (25 μM), glutamate (10 mM) and malate (4 mM). The two‐phase kinetics were observed even when succinate was not present. [file JCSM-16-e13775-s002.pdf]
